# The slow photo-induced CO_2_ release of *N*-phthaloylglycine†

**DOI:** 10.1039/d4sc01604a

**Published:** 2024-05-24

**Authors:** Wiebke Haselbach, Oliver Nolden, Nadine Blaise, Tom Förster, Mick Gindorf, Mathieu Kippes, Michelle P. Rademacher, Matthias Jantz, Luuk J. G. W. van Wilderen, Jens Bredenbeck, Josef Wachtveitl, Peter Gilch

**Affiliations:** a Institut für Physikalische Chemie, HHU Düsseldorf Universitätsstr. 1 40225 Düsseldorf Germany gilch@hhu.de; b Institut für Physikalische und Theoretische Chemie, Goethe Universität Frankfurt Max-von-Laue-Str. 7 60438 Frankfurt/Main Germany; c Institut für Biophysik, Goethe Universität Frankfurt Max-von-Laue-Str. 1 60438 Frankfurt/Main Germany

## Abstract

Carboxylic acids and carboxylates may release CO_2_ upon oxidation. The oxidation can be conducted electrochemically as in the Kolbe synthesis or by a suitable oxidant. In *N*-phthaloylglycine (PG), the photo-excited phthalimide chromophore acts as an oxidant. Here, the photo-kinetics of PG dissolved in acetonitrile is traced by steady-state as well as time-resolved UV/vis and IR spectroscopy. The experiments provide clear evidence that, contrary to earlier claims, the photo-induced CO_2_ release is slow, *i.e.* it occurs on the microsecond time range. The triplet state of PG is, therefore, the photo-reactive one.

## Abstract 2

As time-resolved infrared spectroscopy shows, the title compound undergoes photo-induced CO_2_ release within microseconds – orders of magnitudes longer than anticipated.

## Graphical abstract

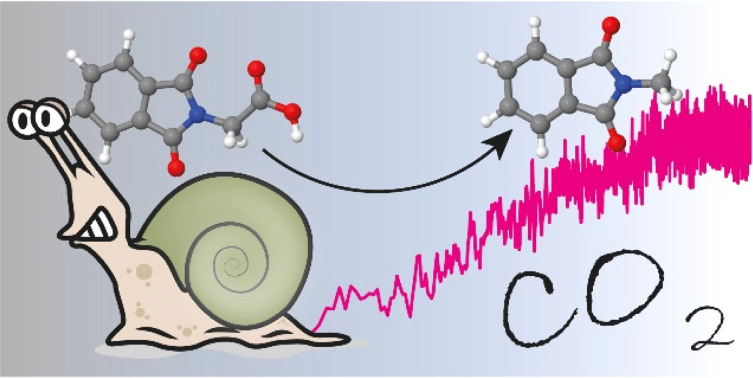

## Introduction

**Scheme 1:**
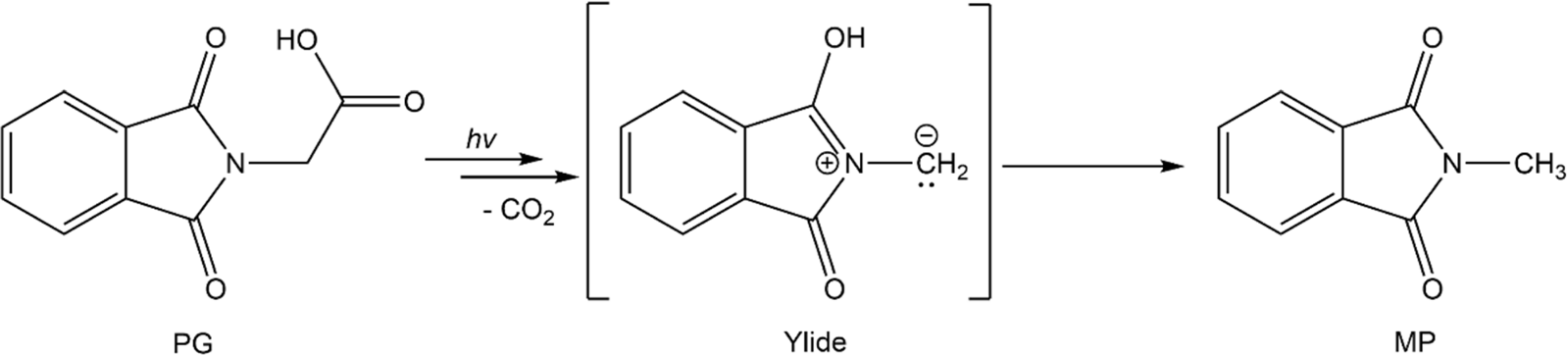
Proposed reaction scheme of PG in CH_3_CN *via* an ylide to form *N*-methylphthalimide.

Carboxylic acids (R–COOH) and carboxylates (R–COO^−^) can release CO_2_ upon H-abstraction and one-electron oxidation, respectively.^1–3^ The radicals R˙ formed thereby can either dimerize yielding R–R or abstract a hydrogen atom resulting in R–H. With this decarboxylation, certain aliphatic and aromatic hydrocarbons are synthetically accessible, for instance from biomass.^3^ In the Kolbe synthesis, the decarboxylation is initiated by an electrochemical oxidation of a carboxylate.^2,3^ The oxidation can also be induced by photo-excitation. In the enzyme photodecarboxylase – first described in 2017 – a photo-excited flavin cofactor oxidizes the carboxylate function of a fatty acid, which is the substrate of this enzyme.^4,5^ The oxidation is a single electron transfer (SET) process occurring with a time constant of ∼300 ps.^5^ The infrared (IR) signature of the released CO_2_ was shown to rise with the same time constant.^5^ The SET process here is, thus, rate determining and no kinetic information on the process R–COO˙ → R˙ + CO_2_ is obtained. This process might be addressable with compounds in which the phthalimide (P) chromophore serves as a light-dependent oxidant.^6,7^ In conjugates of P and –COOH/–COO^−^ moieties, photo-induced CO_2_ release was observed and employed in the organic synthesis of ring systems.^8,9^ The simplest representative of this group is the compound *N*-phthaloylglycine (PG, Scheme 1). Upon UV-irradiation, PG in its protonated (–COOH)^10^ and deprotonated form (–COO^−^)^11^ releases CO_2_ and transforms into *N*-methylphthalimide (MP). The photo-reaction of PG was traced by nanosecond UV/vis absorption spectroscopy.^12^ In that study, a transient with an absorption maximum at 392 nm was observed 200 ns (limited by the instrument) after UV excitation. The transient was assigned to an ylide as depicted in Scheme 1. The postulated presence of this ylide after 200 ns and the observation that P derivatives feature fluorescence lifetimes of nanoseconds or below^13^ suggest that the photo-induced CO_2_ release in PG involves an excited singlet state and might occur within well below one nanosecond. A quantum chemical study is in (partial) support of this singlet mechanism.^14^ In the study, a conical intersection (CI) connecting the S_1_ state of PG with the ylide was identified. Yet, according to computations, a barrier of 0.74 eV has to be surmounted to access this CI.

Here, as part of our efforts to study the Kolbe-type CO_2_ release by means of time-resolved spectroscopy, spectroscopic experiments on the photo-induced CO_2_ release of PG will be presented. To this end, steady-state as well as time-resolved UV/vis absorption and IR spectroscopy were employed. To connect with the spectroscopic study mentioned above,^12^ all experiments were conducted in acetonitrile (CH_3_CN and CD_3_CN). CD_3_CN was employed in some IR measurements for reasons of transmission. In acetonitrile, the protonated form of PG should clearly prevail. According to a recent survey,^15^ carboxylic acids like PG exhibit p*K*_a_-values of ∼11 or higher in acetonitrile. For typical concentrations employed here (∼1 mM), this translates into a degree of dissociation of 10^−4^ or smaller. The experiments show clearly that for protonated PG the CO_2_ release is a microsecond process involving the triplet state of PG.

## Results

### Steady-state UV/vis spectroscopy

**Fig. 1:**
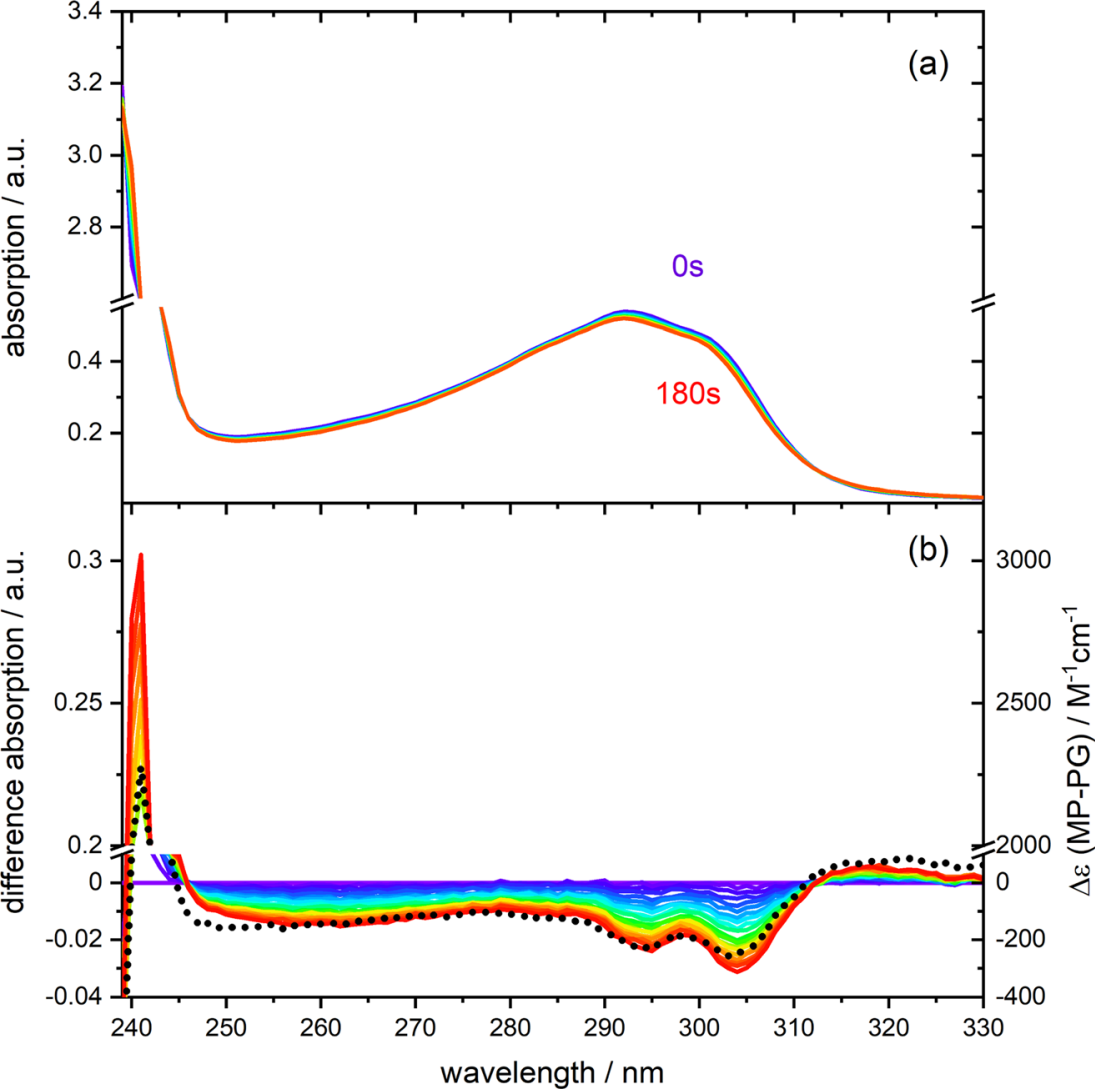
Photo-reactivity of PG (0.30 mM) dissolved in deoxygenated acetonitrile. (a) UV/vis absorption spectra of PG at different times of illumination at 292 nm (0–180 s). (b) Difference spectra (illuminated minus non-illuminated) are derived from the spectra in panel (a). A predicted difference spectrum based on the absorption spectra of MP and PG is shown for comparison (dotted black line, details see Fig. S1†).

The UV/vis absorption spectrum of PG in CH_3_CN features a lowest energy absorption band peaking at 292 nm (absorption coefficient *ε*_292_ = 1830 M^−1^ cm^−1^, see Fig. 1 and S1 in the ESI†). The band is slightly structured presumably due to a vibronic progression and strongly resembles the one of MP.^13^ For MP the peak absorption coefficient is slightly lower (*ε*_292_ = 1625 M^−1^ cm^−1^). The similarity of the bands of PG and MP indicates that they are due to an electronic transition of the same character. A spectroscopic and quantum-chemical study assigned the MP band to a higher excited state S_*n*≥2_ of ππ* character.^13^ A transition of the same character is expected for PG. UV irradiation of PG dissolved in deoxygenated CH_3_CN at 292 nm results in a slight decrease of the absorption around 292 nm. For wavelengths smaller than ∼245 nm and larger than ∼310 nm, the absorption increases slightly. This is easier to discern in the difference representation (Fig. 1(b)). These difference spectra change their magnitude with illumination time but not their shape. This points to a uniform photoreaction. The shape of these spectra matches the one of a difference spectrum obtained by subtracting the PG spectrum from the MP one, *i.e.* Δ*ε*(*λ*) = *ε*_MP_(*λ*) − *ε*_PG_(*λ*). This supports earlier findings that MP is the (predominant) photoproduct of PG in deoxygenated acetonitrile.^10^ However, in aerated acetonitrile, difference spectra obtained by UV illumination differ in shape from the prediction *ε*_MP_(*λ*) − *ε*_PG_(*λ*) (see Fig. S2†). From the initial slope of the absorption *A* at 292 nm *versus* illumination time, the difference absorption coefficient Δ*ε*_292_ and the absorbed light power, the reaction quantum yield *Φ*_r_ was determined (see Fig. S3†). For an initial PG concentration of 3 × 10^−4^ M, and in absence of dissolved oxygen it amounts to 0.4 ± 0.1 (concerning error estimates, see caption to Fig. S2 and S3†). For aerated acetonitrile, a somewhat smaller value of 0.3 ± 0.1 was obtained.

### Steady-state IR spectroscopy

**Fig. 2:**
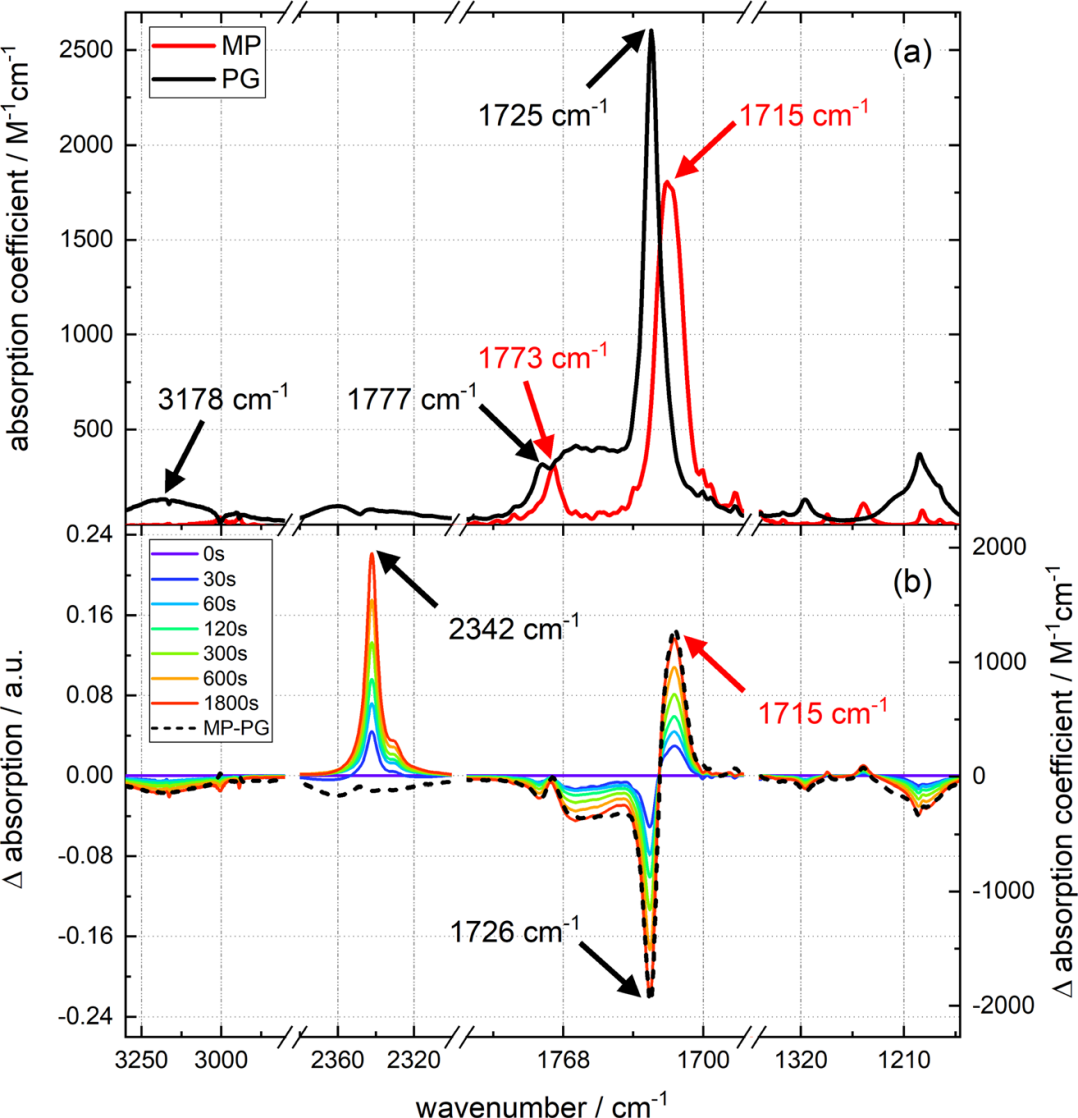
Steady-state IR spectroscopy of the PG photochemistry. (a) IR spectra (absorption coefficient *versus* wavenumber) of PG and MP dissolved in acetonitrile (CH_3_CN). The solvent contribution to the IR spectra was subtracted. Axis breaks denote spectral regions which are opaque due to solvent absorptions. (b) Photoinduced changes of the IR absorption of a solution of PG (12.3 mM) in deoxygenated acetonitrile (CH_3_CN). The excitation wavelength was 292 nm. The difference spectra are obtained by subtracting the PG spectrum from the spectra at indicated illumination times. A predicted difference spectrum based on the IR spectra of PG and MP is shown for comparison. Characteristic wavenumbers are indicated by black (PG) and red (MP) arrows.

The strongest IR resonances of PG and MP dissolved in acetonitrile (CH_3_CN) are located around 1700 cm^−1^ (Fig. 2) where C=O stretching vibrations^16^ are located. In MP, the band at 1773 cm^−1^ can be assigned to the symmetric stretching vibration of the carbonyls within the imide moiety (see red spectrum in top panel of Fig. 2).^17^ The stronger band at 1715 cm^−1^ is the anti-symmetric one. In PG, the respective bands are found at 1777 cm^−1^ and 1725 cm^−1^. The broad band at 1762 cm^−1^ is due to the C=O stretching vibration of the –COOH group.^18^ The O–H stretching vibration of this group peaks at 3178 cm^−1^. As carboxylic acids tend to form hydrogen-bonded dimers,^19^ and the IR experiments were conducted at high concentrations (up to 73 mM for time-resolved IR experiments), the concentration dependence of the PG IR spectrum was investigated (see Fig. S4†). For concentrations up to ∼70 mM, the IR signal scales linearly with the concentration. Thus, hydrogen-bonded dimers do not seem to play a role here.

Upon illumination of a deoxygenated solution of PG with 292 nm light, pronounced changes in the IR spectrum are observed (see difference spectra in Fig. 2). There is a bleach at 3190 cm^−1^ and a positive band at 2342 cm^−1^. This band can safely be assigned to the anti-symmetric stretching vibration of CO_2_ dissolved in acetonitrile.^20,21^ In the carbonyl stretching region, there are bleaches at higher wavenumbers and a positive band at 1715 cm^−1^. In the fingerprint region, several bleaches and positive bands appear. As in the UV/vis absorption experiment, only the signal magnitude and not the spectral shape changes with illumination time. Except for the CO_2_ contribution, the difference spectrum obtained by illumination is well reproduced by the signature obtained by subtracting the IR spectrum of PG from the one of MP, *i.e.* Δ*ε*(*ν̃*) = *ε*_MP_(*ν̃*) − *ε*_PG_(*ν̃*). From the difference spectra and IR absorption coefficients, the ratio *r*_cp_ between photo-generated CO_2_ and consumed PG can be estimated. This ratio is derived from eqn (1),1
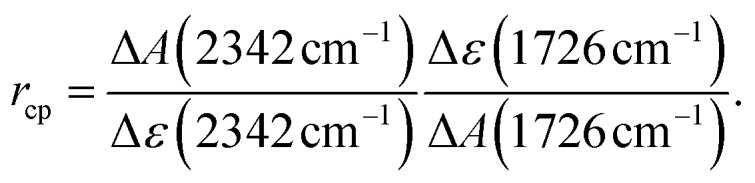
Here, Δ*A*(2342 cm^−1^) is the difference absorption at the maximum of CO_2_ stretching band and Δ*A*(1726 cm^−1^) the one for strongest bleach signal. It is assumed that there is no absorption of MP at 2342 cm^−1^. For an illumination time of 900 s, Δ*A*(2342 cm^−1^) amounts to 0.13 and Δ*A*(1726 cm^−1^) to −0.16. Δ*ε*(2342 cm^−1^) ≈ *ε*(2342 cm^−1^) is the IR absorption coefficient of CO_2_ at the peak of the stretching band. To our knowledge, no IR absorption coefficient of CO_2_ dissolved in acetonitrile has been reported to date. In water this coefficient amounts to 1500 M^−1^ cm^−1^.^22^ For the computation of the ratio *r*_cp_ this value was relied on. The difference absorption coefficient Δ*ε*(1726 cm^−1^) for the bleach at 1726 cm^−1^ was determined to be −1940 M^−1^ cm^−1^. With these values a ratio *r*_cp_ of 1.07 results. The ratio being close to one is in line with the stoichiometry of the photoreaction.

A value for the reaction quantum yield *Φ*_r_ was also derived from the IR difference spectra. As in the determination based on the UV/vis spectra (see above), the IR absorption *A*(*ν̃*) was plotted *versus* the illumination time (see Fig. S5†). From the initial slopes of these plots and the respective difference absorption coefficients Δ*ε*(*ν̃*), the quantum yield *Φ*_r_ was computed. For technical reasons, the absorbed light power could not be measured directly. Therefore, this light power was determined with the actinometer *o*-nitrobenzaldehyde dissolved in CH_3_CN.^23^ The values determined for different wavenumbers were averaged and result in a quantum yield of 0.5 ± 0.1 in deoxygenated CH_3_CN. This value is slightly higher than the one inferred from the UV/vis experiment. In the presence of oxygen, the yield is reduced to 0.2 ± 0.1. This is slightly lower than the value from the UV/vis experiment. For analogous measurements in CD_3_CN, we determined values of 0.4 ± 0.1 in deoxygenated and 0.1 ± 0.1 in aerated solvent (see Fig. S6†).

### Time-resolved UV/vis absorption spectroscopy

**Fig. 3:**
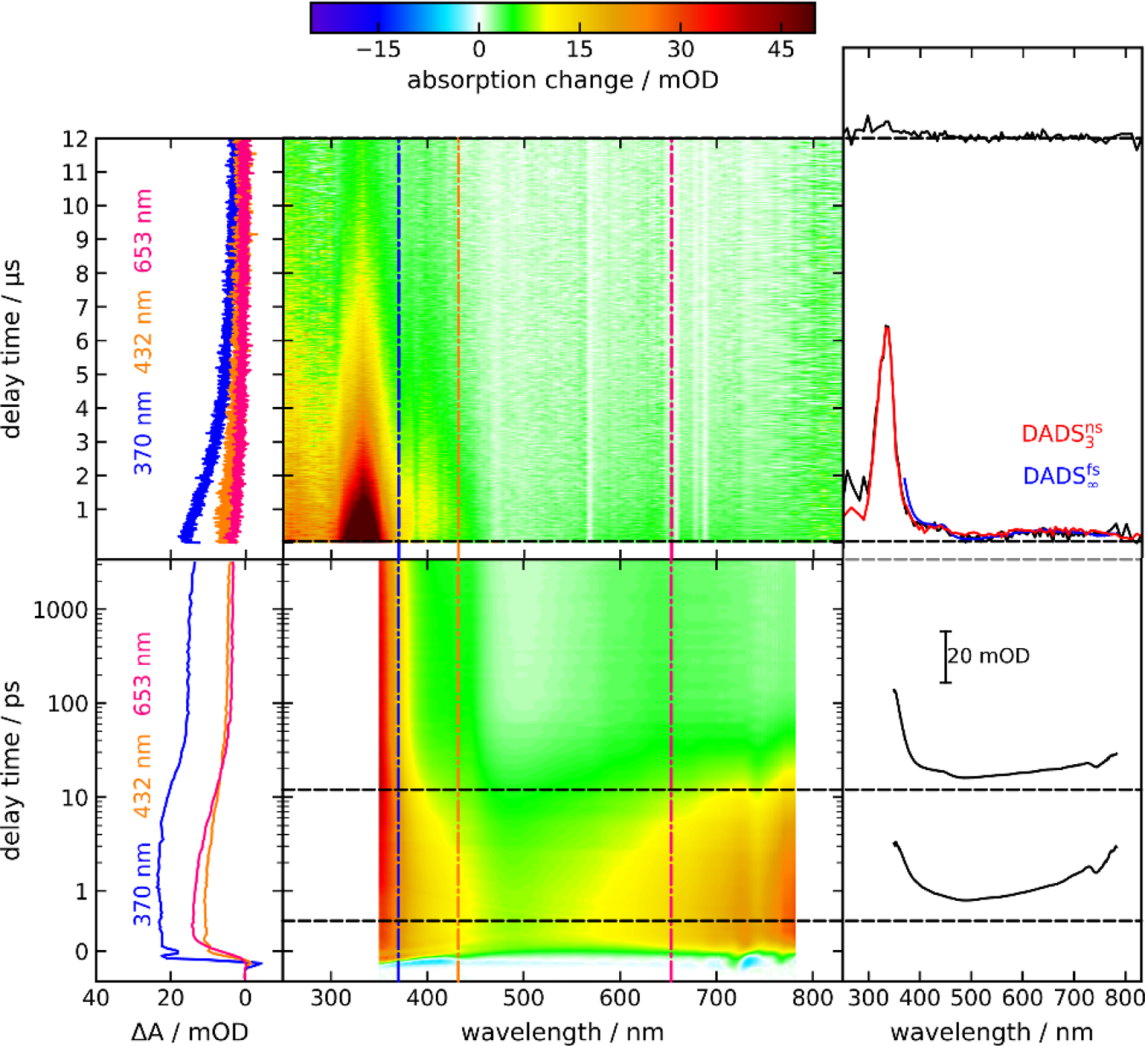
Femtosecond (bottom) and nanosecond (top) UV/vis absorption spectroscopy on PG dissolved in CH_3_CN. In the central contour representation, the difference absorption as a function of detection wavelength and delay time is color-coded. Vertical lines mark spectral positions for the time traces plotted on the left. Horizontal lines mark delay times for the difference spectra plotted on the right. In the femtosecond experiment, the excitation was tuned to 300 nm and the concentration amounted to 3.6 mM. In the nanosecond experiment, the excitation wavelength was 266 nm and the concentration amounted to 1.3 mM. For the nanosecond experiment, deoxygenated acetonitrile was employed. The decay-associated difference spectrum DADS^fs^_∞_ (blue) from the fsTA experiment, corresponding to the offset signature of this experiment, is compared to the transient spectrum in the nsTA experiment after ∼50 ns and DADS^ns^_3_ (red). Note that the spectral coverage of the nsTA instrument is larger than the one of the fsTA instrument.

By time-resolved UV/vis absorption spectroscopy, an overview of the photo-kinetics of PG is attained. Solutions of PG in acetonitrile (CH_3_CN) were excited with femtosecond pulses centered at 300 nm. Resulting absorption changes were probed by white light in the range of 350 nm to 770 nm (bottom panel of Fig. 3). Around time zero a strong positive difference absorption signature with a band around 380 nm and an absorption increase towards the near-infrared is observed. Negative signatures due to ground state bleach and stimulated emission are absent. Ground state bleach – if present – ought to peak around 290 nm (*cf.*Fig. 1), a spectral region which was not covered here. Based on the fluorescence spectrum of the phthalimide chromophore,^13^ stimulated emission ought to peak around 400 nm. Yet, the excited singlet state responsible for the fluorescence features a small oscillator strength^13^ and thus a weak stimulated emission. This weak (negative) signal is obviously overcompensated by a stronger excited state absorption. The time zero signature persists for ∼10 ps and then gives way to a spectral pattern with a strongly increasing signal towards the UV and a very weak peak around 640 nm (see Fig. 3, top right-hand panel). This pattern remains constant in shape and amplitude until ∼3 ns (largest accessible delay time). The decay of this pattern was probed by nanosecond UV/vis absorption spectroscopy in the range of 250 nm to 830 nm (top panel of Fig. 3). In the experiment, a deoxygenated PG solution was excited at 266 nm. The earliest spectrum of this experiment (∼50 ns) features a band peaking at 335 nm, a shoulder at 400 nm and a weak band around 650 nm. For the concentration employed (1.3 mM), the signature decays on the time scale of 10 μs. Around 250 nm, a positive difference absorption signature remains after this decay.

**Fig. 4:**
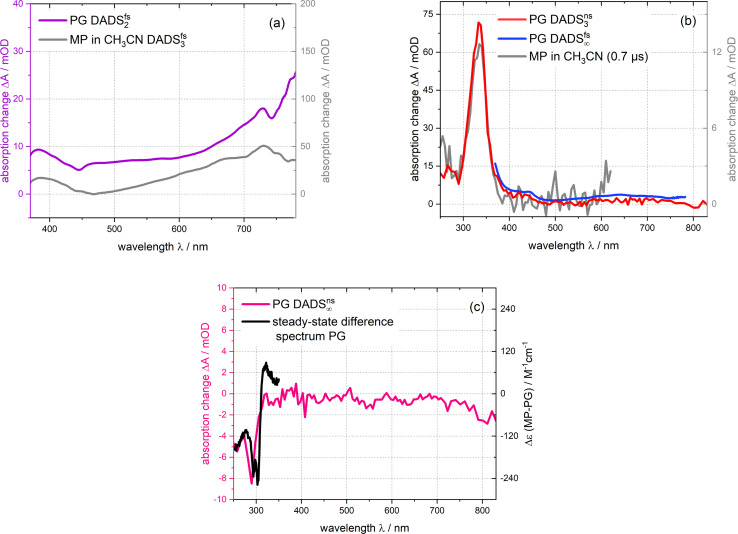
DADS derived from the femtosecond and nanosecond UV/vis absorption measurements on PG dissolved in acetonitrile depicted in Fig. 3. The DADS_2-3_ are compared with the ones of MP^13^ ((a) and (b)). The DADS^ns^_3_ for PG retrieved from the nanosecond experiment is also compared to the offset spectrum of the femtosecond experiment (DADS^fs^_∞_) (b). The offset spectrum of the nanosecond experiment (DADS^ns^_∞_) is plotted together with the steady-state difference spectrum (*cf.*Fig. 1) (c).

Time constants and decay-associated difference spectra (DADS) were retrieved by a global fitting routine with a multi-exponential trial function. Processes with time constants shorter than 100 fs aside, the femto- and nanosecond measurements can be parametrized by only two components. The femtosecond experiment is described by the time constant *τ*_2_ of 15 ps (the numbering of the time constants takes a shorter one in the IR experiment into account) as well as an offset describing processes taking longer than ∼3 ns. The DADS for the time constant *τ*_2_ (DADS^fs^_2_) is spectrally very broad with sigmoidal features at ∼450 and 750 nm (Fig. 4(a)). It is very similar to the one determined for MP in acetonitrile (*cf.*Fig. 4(a)) which was assigned to the decay of lowest singlet excitation (S_1_).^13^ As expected, the DADS for the offset in the femtosecond experiment (DADS^fs^_∞_) concurs with the difference spectrum recorded after ∼3 ns. From the nanosecond experiment, a time constant *τ*_3_ of 2 μs (DADS^ns^_3_) was retrieved. The respective DADS^ns^_3_ and the “late” spectrum of the femtosecond experiment (DADS^fs^_∞_) are identical in shape (Fig. 4(b)). This gives confidence that no kinetic process is missed due to the gap in temporal coverage (3–50 ns) of the two instruments. The DADS^ns^_3_ is characterized by a sharp peak at 333 nm, a shoulder at 400 nm and a broad band peaking at 640 nm. It strongly resembles the respective spectrum for MP (Fig. 4(b)). That spectrum was assigned to the lowest triplet state T_1_ of MP.^13^ It is, thus, likely that in the nanosecond experiment, the T_1_ state of PG is seen to decay. As triplet states are prone to self-quenching, that is quenching by the same type of molecule in the ground state, and oxygen-quenching,^24^ these effects were tested for. By varying the PG concentration in deoxygenated acetonitrile (see Fig. S7†), the bimolecular rate constant *k*_sq_ for self-quenching was determined to be 1.2 × 10^8^ M^−1^ s^−1^. The rate constant *k*_0_ for the intrinsic decay amounts to 3.5 × 10^5^ s^−1^ corresponding to a lifetime of 2.9 μs. Experiments with varying concentrations of dissolved oxygen afforded a bimolecular rate constant *k*_q,O_2__ for oxygen quenching of 1.0 × 10^9^ M^−1^ s^−1^. The offset spectrum of the nanosecond experiment matches the steady state one (Fig. 4(c)). This suggests that the photoproduct is formed after a couple of microseconds latest.

The transient UV/vis signatures of PG described in this section strongly resemble the ones for MP,^13^ suggesting that the same electronic states, namely S_1_ and T_1_, are observed. No indications for a singlet ylide as suggested in ref. 12 were found. The S_1_ lifetime of PG in acetonitrile (∼15 ps) is strongly reduced compared to the one of MP (∼240 ps).^13^ This might be associated with a photochemical reaction of PG, namely CO_2_ release. The time-resolved IR measurements presented below, suggest otherwise.

### Time-resolved IR spectroscopy

**Fig. 5:**
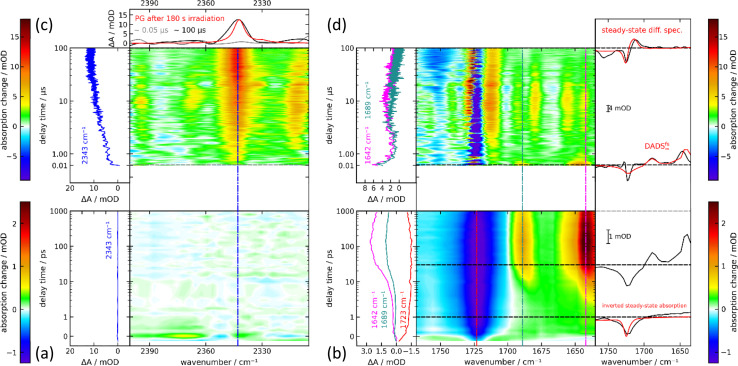
Femtosecond ((a) and (b), dissolved in CD_3_CN) and nanosecond ((c) and (d), dissolved in CH_3_CN) IR spectroscopy on PG. In the central contour representation, the difference absorption as a function of detection wavenumber and delay time is color-coded. Vertical lines mark spectral positions for the time traces plotted on the left. Horizontal lines mark delay times for the difference spectra plotted on the right. (a) and (b): In the femtosecond experiment, the excitation was tuned to 300 nm and the concentration amounted to about 50 mM. (c) and (d): In the nanosecond experiment, the excitation wavelength was 266 nm and the concentration amounted to 73 mM. For the nanosecond experiment, the acetonitrile was deoxygenated.

In the femtosecond IR experiment, PG dissolved in CD_3_CN was excited with a femtosecond pump pulse centered at 300 nm. The induced changes in IR absorption were probed by femtosecond IR pulses. Hereby, the regions around 2350 cm^−1^ (CO_2_ stretching vibration) and 1700 cm^−1^ (carbonyl stretching) were covered. In the region around 2350 cm^−1^, there is a weak bleach contribution centered at 2350 cm^−1^ adjacent to which positive difference absorption signals are detected (Fig. 5(a)). These signals are observed around time zero and only persist for the duration of the instrumental response time. They are presumably mainly caused by cross-phase modulation.^25,26^ Thereafter and up to ∼1 ns, within noise no signal is detected. To ensure that this is not due to a misalignment of the experimental set-up and to define an upper limit of the CO_2_ released up to one nanosecond, a control experiment on *m*-nitrophenylacetic acid was performed. In high pH aqueous buffers, this compound is known to photo-release CO_2_ with a reaction quantum yield *Φ*_r_ of 0.6.^27^ The release was shown to occur within a few 100 ps.^28^ In a femtosecond IR experiment on *m*-nitrophenylacetic acid (*m*NPAA) with settings identical to ones for the PG measurement, the formation of CO_2_ is very clearly observed (see Fig. S8†). After complete CO_2_ release, the IR difference signal at the CO_2_ peak (2342 cm^−1^) amounts to 0.7 mOD. This compares to a noise level of 0.02 mOD as judged from the baseline fluctuations. As the signal measured for *m*-nitrophenylacetate corresponds to a reaction quantum yield *Φ*_r_ of 0.6, the quantum yield of CO_2_ released by photo-excited PG until ∼1 ns cannot be higher than ∼0.02. In the carbonyl region, pronounced IR absorption changes are recorded (Fig. 5(b)). At time zero, there is a strong absorption bleach at 1720 cm^−1^ in line with expectations based on the steady spectrum (*cf.*Fig. 2). The bleach signal is, however, spectrally broader than the respective IR band. In addition, a weak and broad positive signature from 1700–1630 cm^−1^ is recorded. Within ∼10 ps, bands at 1690 and 1640 cm^−1^ rise simultaneously and persist thereafter until ∼1 ns. The 10 ps process does not affect the ground state bleach at 1720 cm^−1^, which remains essentially constant throughout the whole time range.

The IR difference spectrum at ∼1 ns in the carbonyl range differs strongly from the steady state difference (Fig. 2). This and the absence of a CO_2_ signal show that important parts of the PG photochemistry occur on time scales longer than 1 ns. These time scales were covered by step scan FTIR spectroscopy (Fig. 5(c) and (d)). In the respective experiment, a solution of PG in deoxygenated CH_3_CN was excited with 266 nm nanosecond laser pulses. A rather high PG concentration of ∼70 mM was employed. Due to the small optical path length of the IR cell (105 μm) with smaller concentrations a reasonable absorption at the excitation wavelength cannot be attained. With this concentration, the triplet decay ought to be dominated by self-quenching. For the earliest delay time in the nanosecond experiment (∼50 ns), two positive bands at 1645 and 1689 cm^−1^ are observed in the carbonyl range (Fig. 5(d)). A negative band (ground state bleach) is detected at 1725 cm^−1^. This signature overlays favorably with the offset spectrum of the femtosecond experiment suggesting that the same species is observed. It decays within less than 1 μs. Thereafter, in addition to the bleach contribution a spectrum with several bands is recorded persisting from ∼1 μs until ∼30 μs. Except for an additional negative band at 1755 cm^−1^, at 100 μs, a difference spectrum very similar to the steady state one is observed. This implies the photoproduct, namely MP, is formed after 100 μs or earlier. In the CO_2_ stretching region, a very weak if any signal is recorded at ∼10 ns (Fig. 5(c)). Within ∼10 μs it increases to a constant level. The signature is centered at 2343 cm^−1^ and is in accordance with the respective pattern of the steady-state experiment. This gives unequivocal evidence that the CO_2_ release is traced here. An estimate of the reaction quantum yield based on the ground state bleach at early and late delay times is 0.59. This is consistent with the steady-state results.

**Fig. 6:**
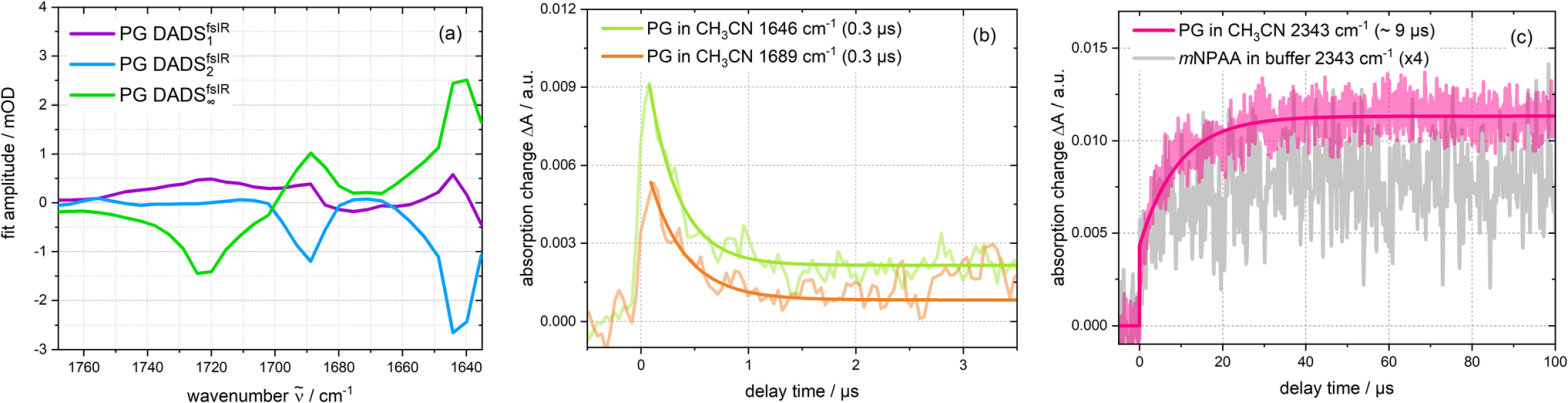
Analysis of the time-resolved IR experiments depicted in Fig. 5. (a) DADS derived from the femtosecond one. (b) Time traces of the nanosecond triplet signature (1646 cm^−1^ and 1689 cm^−1^). (c) Smoothed time trace of the formed CO_2_ band in the nanosecond range for PG in deoxygenated acetonitrile and *m*NPAA (scaled with a factor of 4) in deoxygenated sodium phosphate buffer.

The femtosecond time-resolved IR measurement was also subject to a global analysis (Fig. 6(a)). The time constants retrieved were *τ*_1_ = 1.8 ps (DADS^fs^_1_), *τ*_2_ = 16.7 ps (DADS^fs^_2_) and an offset (DADS^fs^_∞_) describing processes taking longer than ∼2 ns. The time constant *τ*_1_ is longer than the IRF and the corresponding decay-associated difference spectrum (DADS) DADS^fs^_1_ shows only minor spectral changes that may occur within the S_1_ state due to small structural and/or dielectric relaxation after photo-excitation.^29^ The DADS^fs^_2_ shows negative amplitudes at about 1644 and 1689 cm^−1^, indicating a signal increase there. The time constant is consistent with the value (*τ*_2_ = 15 ps) of the femtosecond UV/vis absorption experiment on PG and is assigned to the decay of the S_1_ state. The offset DADS^fs^_∞_ then ought to be assigned to the T_1_ state and matches the “early” measurement of the nanosecond range (Fig. 5). The triplet decay for the respective bands within ∼0.3 μs concurs with expectations based on concentration quenching (0.1 μs) (*cf.*Fig. 6(b)). The CO_2_ rise with 9.4 μs is much slower than the triplet decay (Fig. 6(c)). In a nanosecond control experiment on *m*NPAA acid with similar settings, the formation of CO_2_ is observed without a pronounced delay compared to the PG measurement (see Fig. 6(c) and S8†).

### Quantum chemical calculations

**Fig. 7:**
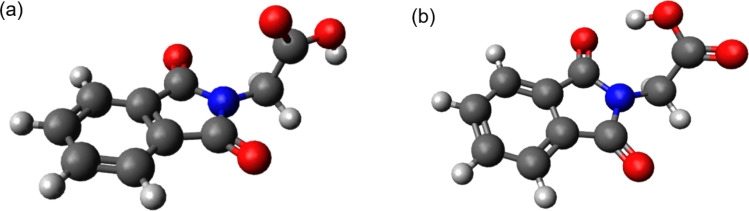
Optimized ground state geometries of PG; (a) open conformer and (b) closed conformer (intramolecular hydrogen bond). The conformers were modelled in acetonitrile according to the polarizable continuum model (PCM) at the B3LYP/Def2-TZVP level of theory.

In the femtosecond IR experiment, a species with resonances in the carbonyl range at 1642 and 1689 cm^−1^ and a rise time *τ*_2_ of 17 ps was detected. In the femtosecond UV/vis experiment, the spectral signature assigned to the triplet state also appears with this time constant. To give further support to this assignment, the IR signature of the PG triplet was computed quantum chemically. PG in the electronic ground state was first geometry optimized. As an intramolecular hydrogen bond was suggested for PG,^30^ structures without such a hydrogen bond (“open” in Fig. 7(a)) and with (“closed” in Fig. 7(b)) were computed (see ESI Table S1†). The computation (B3LYP/Def2-TZVP, a harmonic scaling factor of 1.0044 (ref. 31) was employed) was carried out with Gaussian16.^32^ The solvent acetonitrile was considered implicitly using the polarizable continuum model (PCM, SCRF method). The Gibbs free energy of the open conformer is slightly smaller than the one of the closed forms by 31 meV. Based on this small difference, it cannot be decided which conformer prevails. X-ray crystallography seemingly favors the open form.^33,34^ However, PG forms dimers in the crystal, in which these carboxylic acid moieties form intermolecular hydrogen bands. This could suppress intramolecular hydrogen bonds. Therefore, both conformers might be present in solution, hence the IR signatures of both forms were computed.

**Fig. 8:**
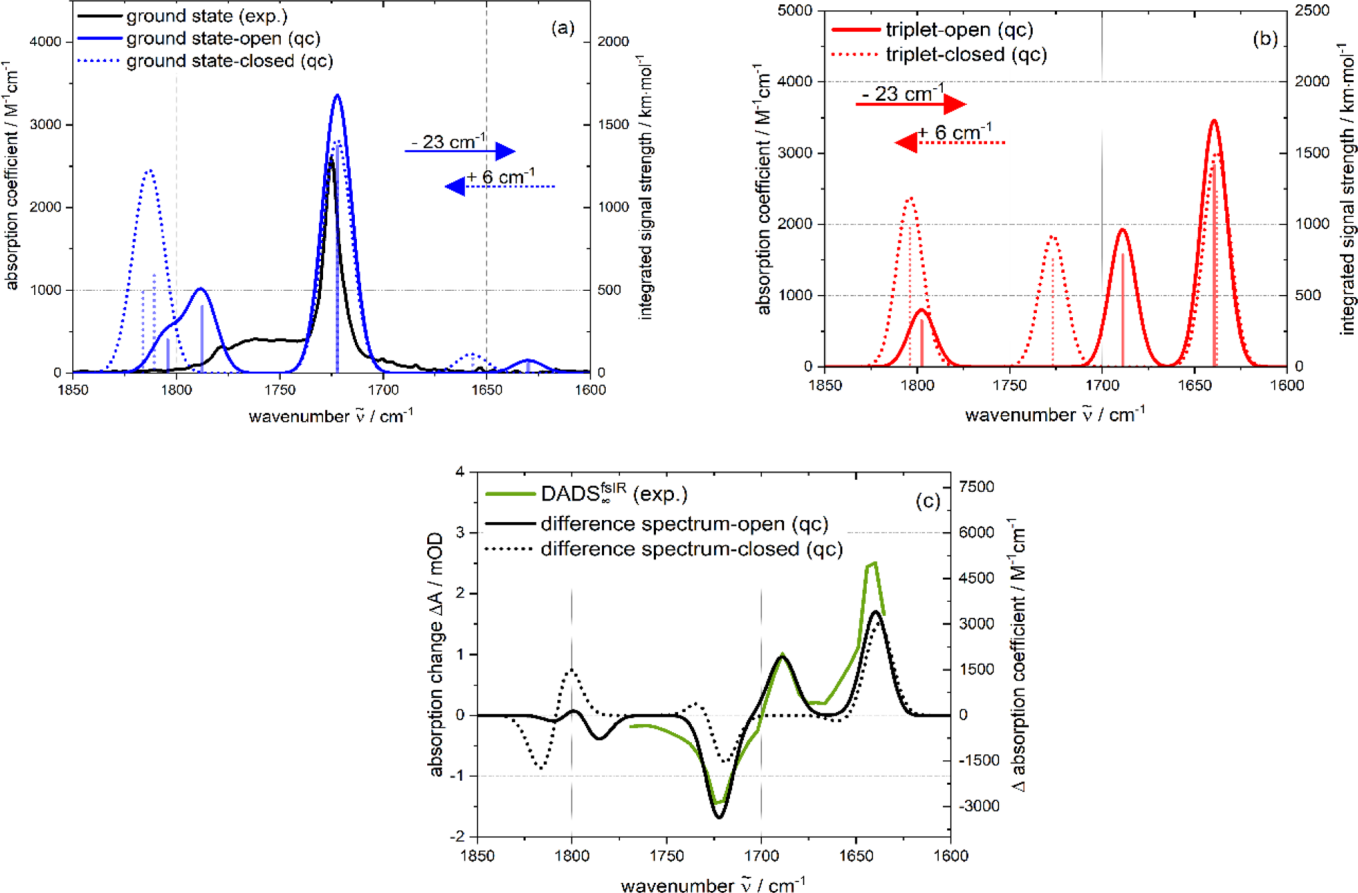
Computed IR spectra of the open (closed) PG conformer (a harmonic scaling factor of 1.0044 (ref. 31) was applied to all calculated frequencies): (a) ground state: comparison of the quantum chemical stick spectrum (blue; open: solid; closed: dotted) (integrated signal strength as a function of wavenumber) convoluted with Gaussians (blue; open: solid; closed: dotted) (absorption coefficient as a function of wavenumber) with the experimental (black) IR spectrum. (b) Lowest triplet state: computed spectra of the open (red solid line) and closed (red dotted line) form. (c) Difference spectra (black; open: solid; closed: dotted), obtained by subtracting triplet from the ground state spectra are compared with the DADS^fsIR^_∞_ (green solid line). The calculated ground and triplet states in (a) and (b) as well as the difference spectra of (c) were shifted along the wavenumber axis to match the negative peak at 1722 cm^−1^ (open: −23 cm^−1^; closed: +6 cm^−1^; indicated by colored arrows in (a)).

For comparison with the experimental spectra (*cf.*Fig. 8(a) and (c)), the quantum chemical stick spectra (integrated signal strength in km mol^−1^) were convoluted with Gaussians (FWHM ∼17 cm^− 1^). With this procedure, also IR absorption coefficients *ε*(*ν̃*) as a function of the wavenumber *ν̃* were computed. In general, the computation reproduces the experimental absorption coefficients rather well. To facilitate the comparison, the computed spectra were shifted by −23 cm^−1^ (open form) and +6 cm^−1^ (closed form) in Fig. 8. The computed wavenumbers given in the text refer to unshifted values. The relevant parts of the computed spectra are compared with the experimental ones (*cf.*Fig. 8). For the ground state of PG, the computation for the open conformer predicts a weak band at 1653 cm^−1^ due to a stretching vibration of the phenyl ring. Such a band is not observed in the experimental spectrum. The strong band at 1745 cm^−1^ is due to the anti-symmetric CO stretching vibration of the imide moiety. According to the computations, the band at 1810 cm^−1^ has a predominant imide symmetric CO stretching character. The one at 1827 cm^−1^ is predominantly CO stretching of the –COOH moiety. As a comparison of the experimental IR spectra of MP and PG shows (*cf.*Fig. 2), the wavenumber of this mode is in between the ones of the CO vibration of the imide. However, the relative strength compares favorably with the experimental values. For the closed conformer, the stretching vibration of the phenyl ring is predicted at 1651 cm^−1^. The two CO stretching vibrations of the imide function are placed at 1716 cm^−1^ and 1810 cm^−1^, the CO stretching of the –COOH moiety at 1805 cm^−1^. The ratio of the relative strengths of the predicted bands differs from the experiment. Thus, computations for both conformers are partially in conflict with the experiment. The computed pattern for the imide vibrations is in better agreement with the experiment for the open form.

According to the computation of the open (closed) conformer, promoting PG to the T_1_ state shifts the two imide CO stretching vibrations to lower wavenumbers, 1662 cm^−1^ (1632 cm^−1^) for the anti-symmetric and 1712 cm^−1^ (1720 cm^−1^) for the symmetric ones (*cf.*Fig. 8(b)). This is expected since the electronic excitation should mainly alter the electronic density on the phthalimide moiety (see electron density difference maps, Fig. S9†). The CO stretching band of the –COOH moiety is only weakly affected by excitation. By subtracting the computed PG IR spectrum of the ground state from the one of the T_1_ state, a difference spectrum was generated and compared with experimental IR DADS^fsIR^_∞_ (Fig. 8(c)). According to the above argumentation, the T_1_ state should be the carrier of this experimental difference spectrum. It is well reproduced by the computation of the open conformer. In the computation, two position bands of 1662 cm^−1^ and 1712 cm^−1^ due to the excited imide moiety are observed. For the closed form, only one band appears (1632 cm^−1^). The agreement for the open form gives further support that in the femtosecond IR experiment the T_1_ state of PG is observed.

## Discussion

In this study, it was confirmed that PG in its protonated form is highly photo-reactive. In the absence of oxygen, the reaction quantum yield *Φ*_r_ is ∼0.4 ± 0.1. The photo-reaction was shown to be “clean” with MP and CO_2_ as the predominant products. Dissolved oxygen lowers the yield *Φ*_r_ and causes the formation of additional – unidentified – products. In the femtosecond experiments, PG is promoted to a higher excited singlet state S_*n*≥2_ of ππ* character (see above). Based on findings on MP, PG is expected to transition to the S_1_ state within less than 100 fs^13^ and thereby within the IRF times of the experiments. The S_1_ lifetime of PG (*τ*_2_ = 15–17 ps) is strongly reduced compared to its non-reactive counterpart MP (∼240 ps). This lifetime reduction is not due a reactive channel open only for PG. After the decay of the S_1_ state, no signature of the photo-products is observed. In particular, the femtosecond IR experiment excludes CO_2_ release in the time window up to a few nanoseconds. In PG and MP, the S_1_ decay is accompanied by the population of the lowest triplet state *T*_1_. Thus, it seems likely that the decreased S_1_ lifetime in PG is associated with an accelerated intersystem crossing (ISC). This accelerated ISC is surprising in the light of results on MP.^13^ MP dissolved in water exhibits a much longer S_1_ lifetime (∼3 ns) than in acetonitrile (∼240 ps). With the aid of quantum-chemical computations, this was attributed to an energetic up-shift of a ^3^*n*π* state by hydrogen bonding and a concomitant decrease of the ISC rate constants. The fact that in the aprotic solvent, PG does not feature a longer S_1_ lifetime gives support to the absence of an intramolecular hydrogen bond addressed above. This leaves the question of why the S_1_ lifetime is strongly reduced. At present, a definite answer cannot be given. We speculate that the –CH_2_–COOH group of PG slightly shields the phthalamide moiety from the polar solvent acetonitrile. For MP, an apolar surrounding (cyclohexane) causes an S_1_ lifetime of 13 ps,^13^ close to the one of PG in acetonitrile. To substantiate this interpretation further spectroscopic and quantum chemical investigations are necessary. Regardless of the background of the accelerated ISC, the femto- and nanosecond experiments clearly show that the T_1_ state of PG is the reactive one. The nanosecond UV/vis and IR experiments indicate that the triplet decay is associated with the formation of the photoproducts MP and CO_2_. Contrary to the femtosecond experiment, CO_2_ was detected in the nanosecond IR experiment. Thus, earlier claims by Takahashi *et al.* that the reaction proceeds *via* the S_1_ state and a singlet ylide can be considered disproved.^12^ This finding is in line with a quantum chemical study, which found a pathway between the S_1_ state and the singlet ylide.^14^ Yet, it involved a barrier of 0.74 eV. With such a high barrier, a reaction within the S_1_ lifetime can safely be excluded.

**Scheme 2:**
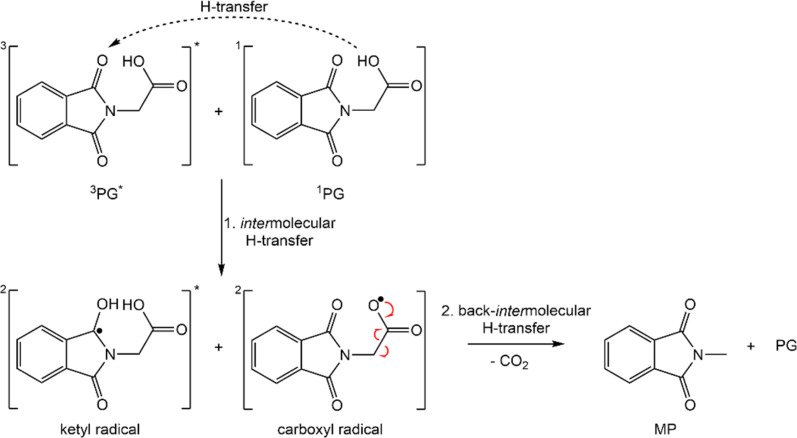
Intermolecular hydrogen transfer between ground state PG and triplet PG.

The finding that the photo-reaction proceeds *via* the T_1_ state poses a couple of questions – not all of them can be answered at the present stage. An earlier work by Reiffers *et al.* gave a triplet lifetime of approximately 10 μs for MP in acetonitrile (comparable concentrations).^13^ This is a significantly longer lifetime of the triplet state compared to PG in acetonitrile. This reduction in lifetime is in line with the photoreaction contributing to the T_1_ decay in PG. However, like many triplet states,^13,35,36^ the one of PG was shown to be subject to oxygen and concentration quenching. For diluted solutions, the T_1_ lifetime decreases from 2.9 μs in a deoxygenated sample to 0.5 μs in the presence of air. For oxygen quenching competing with the reactive decay, the reaction quantum yield *Φ*_r_ ought to decrease by a factor of 0.5 μs/2.9 μs = 0.16. In the experiment, a factor of ∼0.7 is observed. Thus, also the oxygen quenching pathway – partially – leads to photo-products. The observation that in the presence of oxygen other species in addition to MP are formed is in line with this. Also, the effect of self-quenching is puzzling. For technical reasons, the nanosecond IR experiments had to be conducted with PG concentrations (∼70 mM) for which self-quenching dominates the triplet decay. Under these circumstances, the triplet lifetime amounts to only 0.3 μs. The reaction quantum yield *Φ*_r_, however, remains on the level determined for diluted solutions. This implies that the species formed during self-quenching can transform into MP and CO_2_. Tentatively, this is associated with an intermolecular hydrogen transfer involving triplet PG and ground state PG (Scheme 2). The process would transform the ground state PG into a carboxylate radical and the triplet PG would become a ketyl radical. The formation of the two radicals could then be ensued by the CO_2_ release of the carboxylate radical. The sum of the computed IR signatures of the radicals compares favorably with the experiment (see Fig. S10†). By intermolecular back transfer of the hydrogen atom, a PG and an MP molecule could be formed. This mechanism could explain that the CO_2_ forms with a characteristic time (∼9 μs), which is much longer than the one for the triplet quenching (0.3 μs).

Unfortunately, concerning the intramolecular transformation of triplet PG into MP and CO_2_, the experiments give little information except for the fact that the triplet state is the reactive one. At present, it cannot be decided, whether the transformation includes an ylide type triplet intermediate or not. To clarify that, a quantum chemical study is presently undertaken.

## Conclusion

**Fig. 9:**
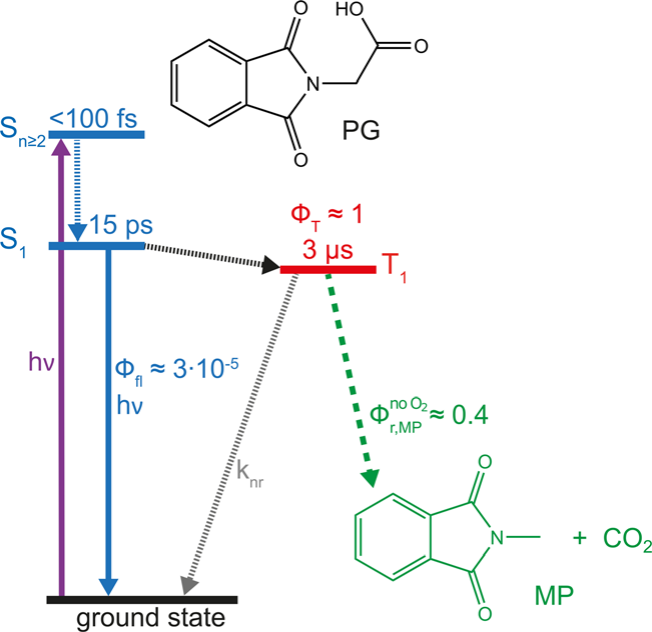
Kinetic scheme of the PG photo-reaction in acetonitrile.

UV excitation of PG in acetonitrile promotes the molecule to the S_2_ state, which decays within less than 100 fs (Fig. 9). Subsequently, the populated S_1_ state persists for 15 ps and decays predominantly by ISC. The fs-IR result presented here excludes the CO_2_ release associated with the ISC process. The reactive state, therefore, has to be the T_1_ state. The CO_2_ release of this state is evident from the ns-IR experiment. However, the observed release is predominantly due to the intermolecular process.

## Experimental

### Samples and general conditions

PG was purchased from ChemScene (99.92%), MP (>99.0%) from Tokyo Chemical Industry, 3-nitrophenylacetic acid (*m*NPAA, 99.0%) from Sigma Aldrich, *o*-nitrobenzaldehyde (≥99.0%) from fluka analytical, acetonitrile (HPLC gradient grade, ≥99.9%) from Carlo Erba, deuterated acetonitrile (99.8%) from Eurisotop, nitrogen (99.999%), argon (99.999%) as well as oxygen (99.999%) from Air Liquide. All chemicals were used as supplied. All measurements were performed at room temperature (19–21 °C). Time-resolved measurements (UV/vis and IR) in the nanosecond to microsecond range were generally performed under deoxygenation (nitrogen) unless described otherwise.

### Steady-state UV/vis spectroscopy

All absorption spectra in the UV/vis were obtained with a two-beam UV/vis/NIR spectrometer from PerkinElmer (Lambda 19). Fused silica cells (Hellma Analytics) with a path length of 10 mm or 1 mm were used. An LED (Roithner Lasertechnik) emitting at 292 nm with 2 mW was used for the irradiation experiments. Aerated and deoxygenated sample solutions were investigated in a 10 mm cuvette with a self-sealing septum and magnetic stirring to determine the reaction quantum yield. Sample volumes of 3 mL were stirred during irradiation.

### Steady-state IR spectroscopy

Absorption spectra in the IR were obtained with an FT-IR-spectrometer from Bruker Optics (Vertex 80v). After passing through the sample, the transmitted IR-light from a globar (silicon carbide) passed an IR longpass filter (transmission 4000 to 900 cm^−1^) and was recorded by a deuterated triglycine sulfate (DTGS) detector from Bruker Optics. The sample compartment was purged by argon. A custom-made flow-through cuvette with CaF_2_ windows of 3 mm thickness and 32 mm diameter (BIOZOL) and a 105 μm Teflon spacer was used. For the irradiation experiments an LED (Roithner Lasertechnik) emitting at 292 nm was used. For the actinometric determination of the reaction quantum yield, the sample reservoir was aerated or purged by nitrogen. To determine the reaction quantum yields, the samples were irradiated for defined illumination periods. The sample volume was exchanged after each illumination period by means of a flow system. The small cuvette dimensions did not allow for sample stirring during the illumination. Since this is the case for both the sample and reference measurements, the results are unaffected. *o*-Nitrobenzaldehyde in acetonitrile (*Φ*_r_ = 0.5) was used as a reference.^23^

### Femtosecond transient UV/vis absorption spectroscopy

The femtosecond transient UV/vis absorption setup was described in detail elsewhere.^37–39^ The 300 nm pump pulses were obtained from the output of a Ti:Sa laser amplifier system (Coherent Libra, output 800 nm, repetition rate of 1 kHz, pulse duration of 100 fs (FWHM)). Part of its output was converted by a noncollinear OPA (TOPAS-white, Light Conversion) to 600 nm and subsequently the frequency was doubled in a β-barium borate crystal to obtain 300 nm pulses. The energy per pulse amounted to ∼1 μJ. White light probe pulses were obtained by supercontinuum generation in CaF_2_. Diameters (FWHM) of the pump and probe beams at the sample location amounted to 160 μm and 100 μm, respectively. The relative polarization of pump and probe light was set to the magic angle. Transient spectra were recorded equidistantly between −1 ps and 1 ps on a linear and from 1 ps to 3.4 ns on a logarithmic time scale. For every delay setting, 2000 spectra were recorded and the data were averaged over 4 succeeding delay scans. Raw data were corrected for the chirp and the solvent contribution.^40^ Absorptions of the sample solutions at 300 nm were adjusted to ∼0.6 in a 1 mm cell. For the measurement, a fused silica flow-through cell (custom made, Hellma Analytics) was used with a 1 mm path length.

### Nanosecond transient UV/vis absorption spectroscopy

Nanosecond transient UV/vis absorption data were acquired with a laser flash photolysis spectrometer from Edinburg Instruments (LP980, right-angle geometry). The fourth harmonic (266 nm) of the output of a Nd:YAG laser (Spitlight 600, InnoLas, Germany, repetition rate 5 Hz) served as excitation light (pump pulse). The pulse duration was 12 ns (FWHM). The average pulse energy amounted to 2.4 mJ. The probe light was generated by a pulsed xenon lamp (Osram XBO 150 W/CR OFR). The transmitted probe light was dispersed by a grating monochromator and detected by a photomultiplier to cover the UV/vis (photomultiplier Hamamatsu PMT-900) spectral range. The signal was digitized by an oscilloscope (MDO3022, Tektronix). Signals were recorded between −4 μs and 35 μs on a linear time scale. The kinetic traces at different probe wavelengths were recorded and averaged over 48 acquisitions to obtain time resolved spectra, 64 acquisitions for the self- and oxygen-quenching studies. Fused silica flow-through cells (Hellma Analytics) with a path length of 5 mm in pump and 10 mm in probe direction were used. Absorptions of the sample solutions at 266 nm were adjusted from ∼0.3 to 1.6 in a 1 cm cell. For the self-quenching study, the changes in lifetime as a function of sample concentration were determined at a wavelength of 335 nm. If not mentioned otherwise, sample solutions were purged with nitrogen. For the investigation of oxygen quenching, sample solutions were exposed to air and saturated with oxygen (pressure ∼1 atm).

### Femtosecond transient IR absorption spectroscopy

The time-resolved IR measurements were performed with a Ti:Sa regenerative amplifier (5 W, Mira Legend Elite HE, Coherent, Santa Clara, CA, USA) with a fundamental of 800 nm, a repetition rate of 1 kHz and a pulse duration of 90 fs. For the measurement, two home-built collinear OPAs were used. Signal and idler pulses from an OPA were subjected to different frequency generation (DFG) in AgGaS_2_, resulting in IR probe pulses. The fundamental was sent to a second OPA where the signal (1200 nm) and the doubled fundamental (400 nm) were focused in a BBO crystal to generate 300 nm pump light. The polarization between pump and probe beams was set to the magic angle. The excitation energy was about 500 nJ per pulse. The IR radiation was dispersed by a grating monochromator and recorded in single-shot fashion by an MCT-detector (Mercury–Cadmium–Telluride Detector, Infrared Associates, USA, 2 × 32 pixels). The spectrometer (Triax 180, Horiba) had a spectral resolution of about 5 cm^−1^. The spectra were recorded equidistantly between −0.1 ps and 1 ps on a linear and from −20 ps to −0.25 ps and 1 ps to 1800 ps on a logarithmic time scale. A custom-made flow-through cuvette with CaF_2_ windows (Crystal GmbH) of 100 μm thickness each and a spacer of 100 μm was used.^41^ A total sample volume of 5 mL was utilized. At about 1720 cm^−1^ the absorption was ∼0.7 OD.

### Nanosecond transient IR absorption spectroscopy

Nanosecond transient IR absorption signals were recorded with a step-scan instrument described in more detail elsewhere.^42^ An instrument from Bruker Optics (Vertex 80v) equipped with a step-scan interferometer module was employed. The sample was pumped by 266 nm light obtained by fourth harmonic generation of the output of a Nd:YAG laser (Spitlight 600, InnoLas, Germany, repetition rate 10 Hz) with 12 ns (FWHM) of pulse duration. The Nd:YAG laser is the same as in time-resolved nanosecond transient UV/vis absorption measurements. The average pulse energy amounted to ∼3.5 mJ. A delay generator (DG35) from Stanford Research Systems was used for step-scan triggering and timing of the pump pulse. After the sample, the transmitted IR-light from a globar (silicon carbide) passed an IR longpass filter (transmission 2646 to 1389 cm^−1^) and was detected by a liquid-nitrogen cooled MCT-detector (Kolmar Technologies). The detector is connected to a fast preamplifier and a 14-bit transient recorder board (TRB, Spectrum Germany, M314142). For digitization at the 14-bit TRB, the time resolution was set to 10 ns. The spectra were recorded between 10 ns and 250 μs on a linear time scale. The spectral resolution was 6 cm^−1^. In total, 16 coadditions at each interferogram point were measured. The same custom-made flow-through cuvette (105 μm path length) as in the steady-state experiment was employed. To avoid contributions from the photo-product, a volume of 200 mL was used for the sample solution and flowed through the cell. The absorption per path length of the sample solution at 266 nm were adjusted to ∼0.6 in a 0.1 mm cell.

### Data analysis

For the determination of the reaction quantum yield *Φ*_r_*via* UV/vis spectroscopy, the initial decrease in the concentration as a function of time 
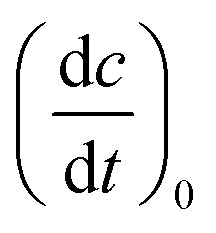
 was used (*cf.*eqn (2)),2
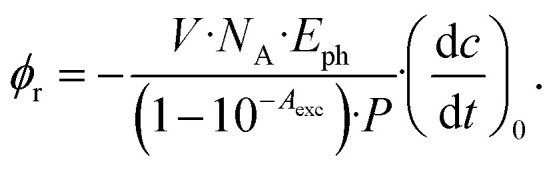
Here, *V* is the sample volume, *N*_A_ the Avogadro constant, *E*_ph_ the energy per photon, *A*_exc_ the absorption at the excitation wavelength of 292 nm, *P* the light power. The initial decrease 
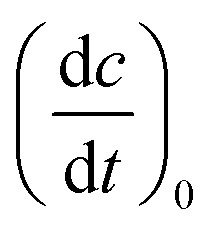
 was determined from the derivative of the difference absorption (Δ*A*/d*t*) *via*eqn (3). The difference absorption coefficient of product and reactant (Δ*ε*) as well as the respective path length (*d*) are considered,3
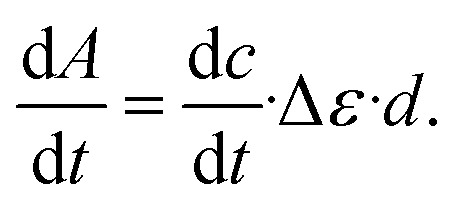


Determinations of the yield *Φ*_r_*via* IR spectroscopy relied on an actinometer (see above). To this end, the initial slopes for the sample 
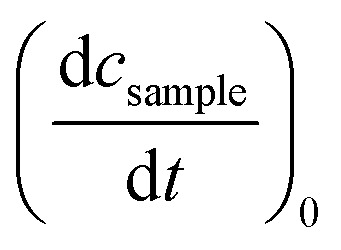
 and the actinometer 
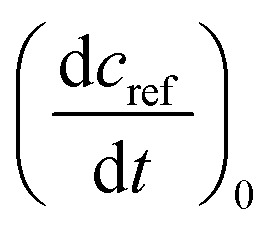
 were compared *via*eqn (4),4
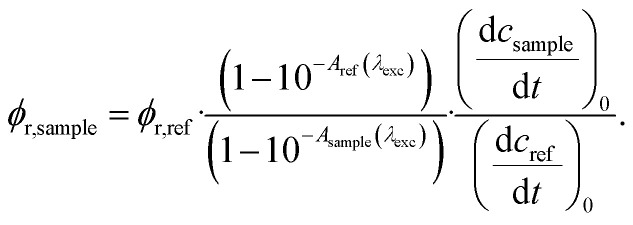
Here, *Φ*_r,ref_ is the reaction quantum yield of the reference, *A*_ref_(*λ*_exc_) the absorption at 292 nm of the reference and *A*_sample_(*λ*_exc_) the absorption at 292 nm of the sample. The slopes 
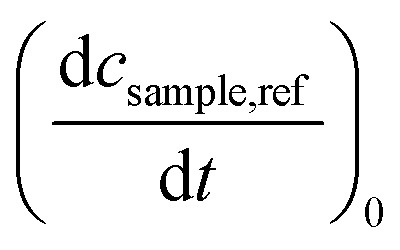
 were determined *via*eqn (3) using IR difference absorption coefficients.

The wavelength-dependent time-resolved transient absorption data (Δ*A*(λ or *ν̃*,*t*)) were analyzed by a global multi-exponential fit function (*cf.*eqn (5)) unless specified otherwise,5
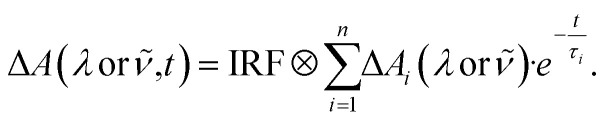


The fit yields a decay-associated difference spectrum (DADS) Δ*A*_i_(λ or *ν̃*) for each time constant *τ*_*i*_.

## Data availability

Data will be made available on reasonable request.

## Author contributions

Nanosecond UV/vis and IR spectroscopy as well as quantum chemical calculations, draft editing, and data visualization W. H.; femtosecond UV/vis spectroscopy as well as draft editing O. N.; femtosecond IR spectroscopy as well as draft editing N. B.; steady-state IR spectroscopy T. F.; steady-state UV/vis spectroscopy M. G.; steady-state IR spectroscopy M. K.; steady-state IR spectroscopy M. P. R.; steady-state IR spectroscopy M. J.; femtosecond IR spectroscopy L. J. G. W. v. W.; supervision of femtosecond IR spectroscopy J. B.; supervision of femtosecond IR spectroscopy as well as draft editing J. W.; conceptualization, supervision of the steady-state and time-resolved spectroscopy, funding acquisition, original draft writing P. G.

## Conflicts of interest

There are no conflicts to declare.

## Supplementary Material

SC-015-D4SC01604A-s001
