# Supplementary material for: The slow photo-induced CO2 release of N-phthaloylglycine
Source: Chem Sci. 2024 May 24;15(25):9719–32. doi: 10.1039/d4sc01604a (PMC11206210; doi:10.1039/d4sc01604a)
Supplement: SC-015-D4SC01604A-s001 [file SC-015-D4SC01604A-s001.pdf]

Electronic Supplementary Material (ESI) for Chemical Science.
This journal is © The Royal Society of Chemistry 2024

Supporting Information

The Slow Photo-induced CO_2 Release of *N*-Phthaloylglycine

Wiebke Haselbach[a,†], Oliver Nolden[a,†], Nadine Blaise[b,†], Tom Förster[a], Mick Gindorf[a], Mathieu Kippes[a], Michelle P. Rademacher[a], Matthias Jantz[a], Luuk J. G. W. van Wilderen[c], Jens Bredenbeck[c], Josef Wachtveitl[b], Peter Gilch[a*]

[a]Institut für Physikalische Chemie, HHU Düsseldorf, Universitätsstr. 1, 40225 Düsseldorf, Germany, gilch@hhu.de

[b]Institut für Physikalische und Theoretische Chemie, Goethe Universität Frankfurt, Max-von-Laue-Str. 7, 60438 Frankfurt/Main, Germany

[c]Institut für Biophysik, Goethe Universität Frankfurt, Max-von-Laue-Str. 1, 60438 Frankfurt/Main, Germany

[†]These authors contributed equally.

1. Steady-State UV/Vis Spectroscopy

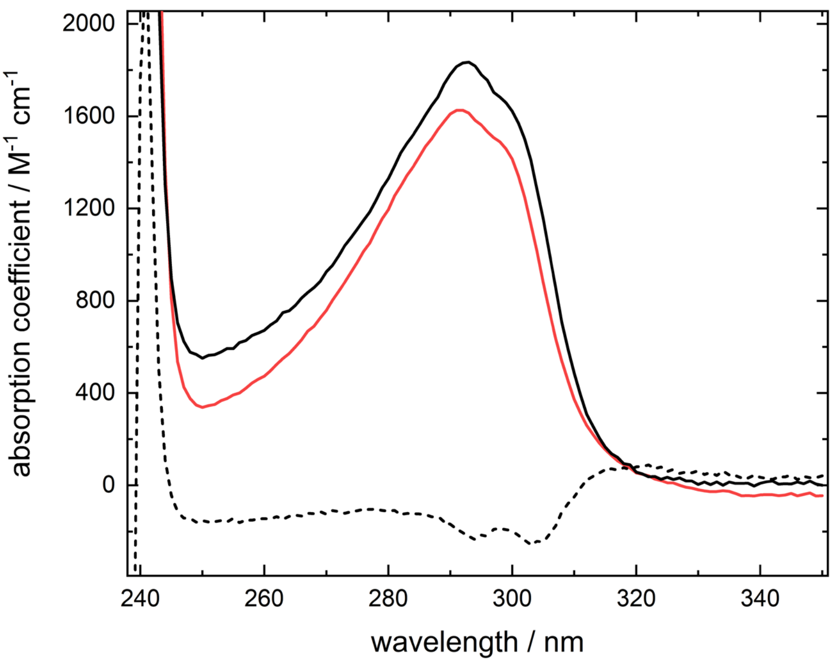

Figure S1. Absorption coefficient spectra of PG (black) and MP (red) and the calculated difference spectrum ($\Delta\varepsilon(\lambda) = \varepsilon_{MP}(\lambda) - \varepsilon_{PG}(\lambda)$, dashed black line). The difference absorption coefficient at 292 nm ($\Delta\varepsilon(292\ \mathrm{nm})$) amounts to 205 $M^{-1}cm^{-1}$ ± 45 $M^{-1}cm^{-1}$. The error estimate is based on a regression analysis using solutions with different concentrations.

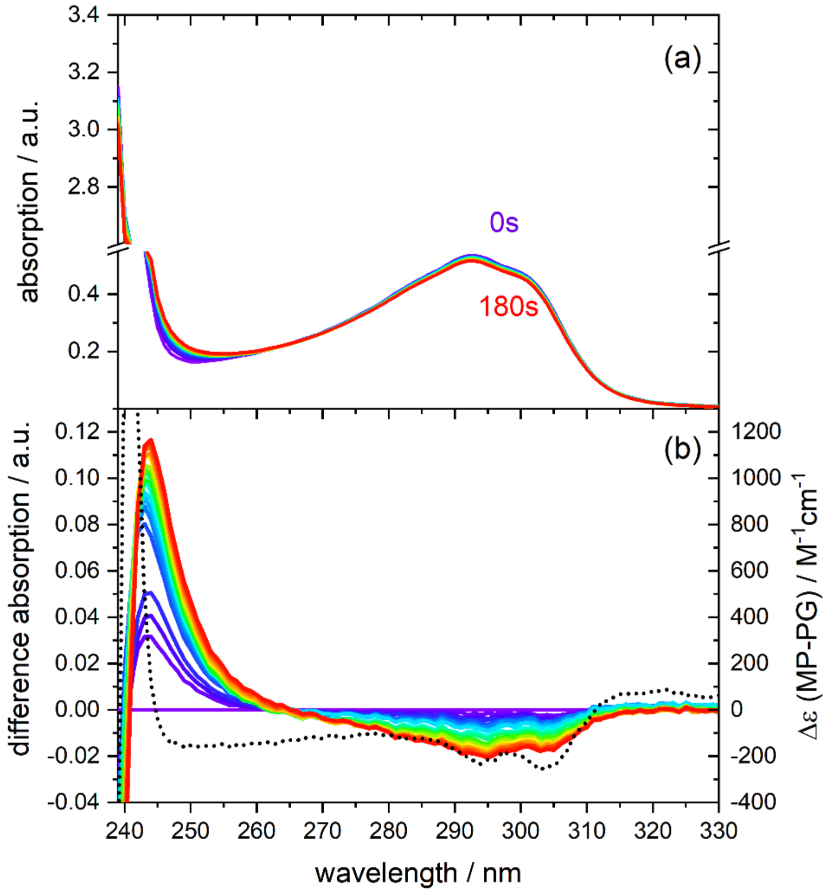

Figure S2. Photo-reactivity of PG (0.29 mM) dissolved in aerated acetonitrile. (a) UV/Vis absorption spectra of PG at different times of illumination (0-180 s) at 292 nm. (b) The difference spectra (illuminated minus non-illuminated) are derived from the UV/Vis spectra for the respective illuminated time. Based on Figure S1, a predicted difference spectrum is shown for comparison (dotted black line). The reaction quantum yield Φ_r amounts to 0.3±0.1. The statistical error is calculated from the uncertainty of the difference absorption coefficient $\Delta\varepsilon(292\ \mathrm{nm})$ from Figure S1 and the uncertainty of the slopes from Figure S3.

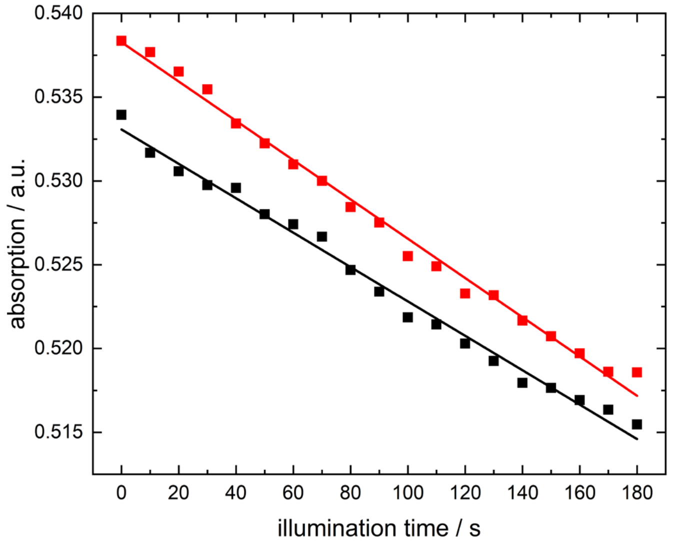

Figure S3. Absorption at 292 nm as a function of illumination time for PG in deoxygenated (red) and aerated (black) acetonitrile. Time traces were extracted from the time-dependent spectra plotted in Figures 1 (deoxygenated) and S2 (aerated). Squares represent the experimental results, lines the results of linear fits. The reaction quantum yields Φ_r were determined using the slopes of these lines. Errors of these slopes and of the difference absorption coefficient $\Delta\varepsilon(292\ \mathrm{nm})$ entered the error estimate of the reaction quantum yields Φ_r.

2. Steady-State IR Spectroscopy

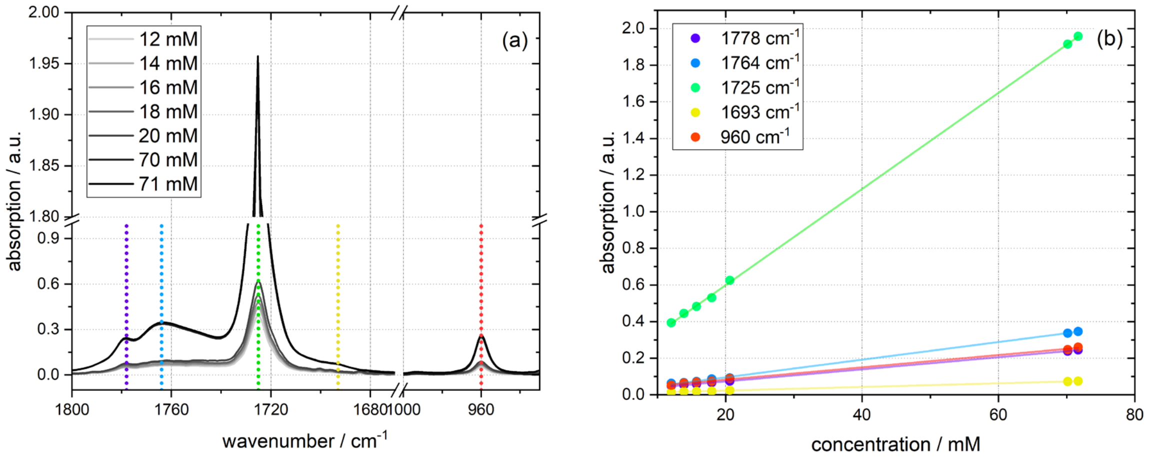

Figure S4. Steady-state IR spectroscopy of PG at different concentrations. The measurements were all carried out with a 105 µm Teflon spacer. Spectra at different concentrations (a) highlight the shape invariance of these spectra. Concentration dependencies at selected wavenumbers (dots: experimental values, lines: linear fit) stress the linear dependence (b). The linear regression performed shows an R^2 term of at least 0.998 for all wavelengths shown.

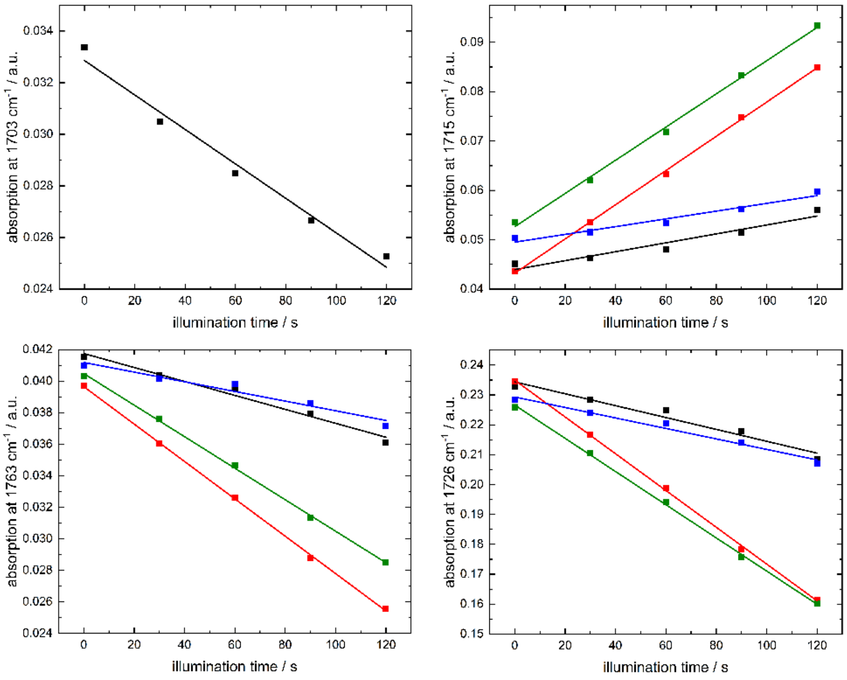

Figure S5. Absorption of *o*-nitrobenzaldehyde at 1703 cm^{-1} (top left) and PG at 1715 cm^{-1} (top right), 1726 cm^{-1} (bottom left) and 1763 cm^{-1} (bottom right) as a function of illumination time. The colors indicate different solvent conditions (black: aerated CH_3CN, red: deoxygenated CH_3CN, blue: aerated CD_3CN, green: deoxygenated CD_3CN). Time traces were extracted from the time-dependent spectra plotted in Figures 2 (deoxygenated CH_3CN) and S6 (aerated and deoxygenated CD_3CN and aerated CH_3CN). Squares represent the experimental results, lines the results of linear fits. The reaction quantum yields Φ_r were determined using the slopes of these lines. The statistical errors of the quantum yields were calculated from the uncertainty of these slopes and the uncertainty of the difference absorption coefficient.

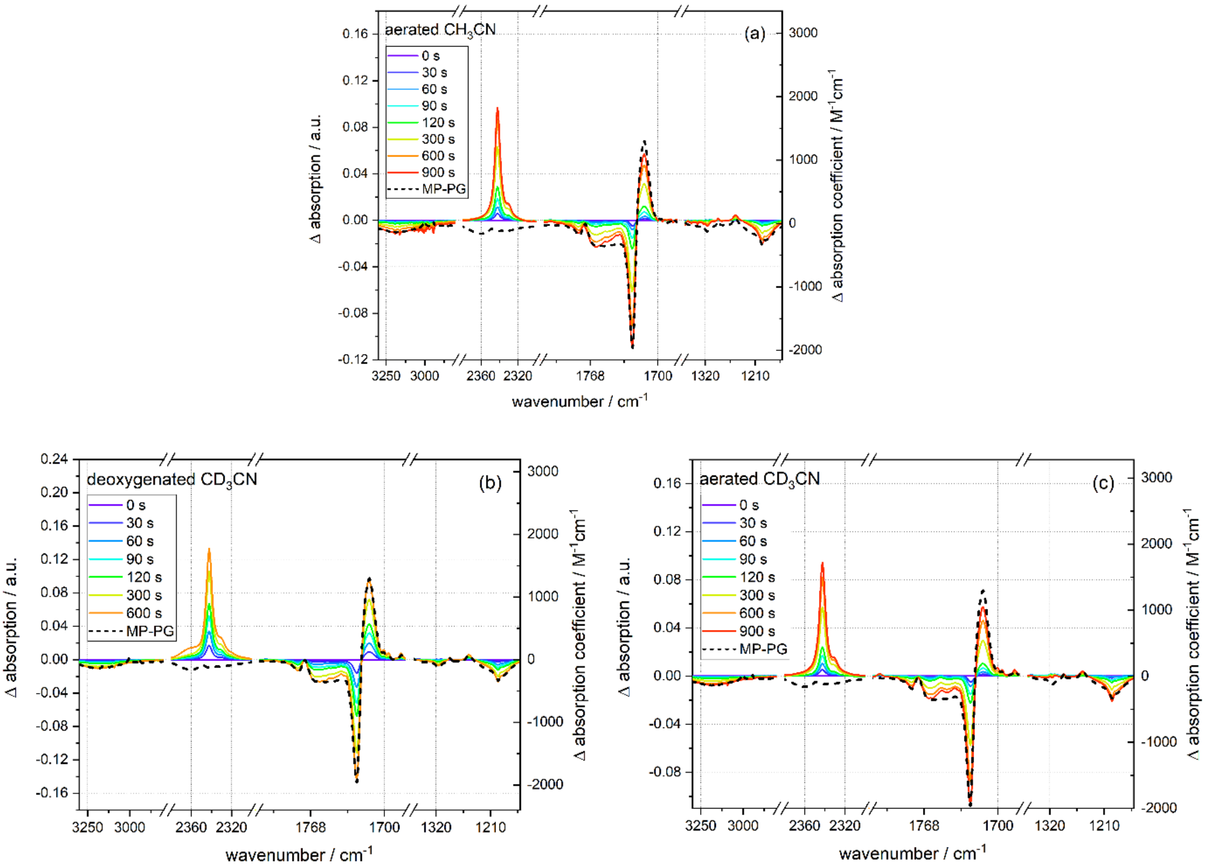

Figure S6. Steady-state IR spectroscopy of the PG photochemistry. Photoinduced changes of the IR absorption of PG in aerated CH_3CN after illumination with 292 nm (~ 11 mM; Φ_r = 0.2±0.1) (a), deoxygenated CD_3CN (~ 9 mM; Φ_r = 0.4±0.1) (b) and aerated CD_3CN (~ 9 mM; Φ_r = 0.1±0.1) (c). The difference spectra are obtained by subtracting the PG spectrum from the spectra at indicated illumination times. The predicted MP-PG difference spectrum is always shown (dashed line).

3. Bimolecular Quenching of the PG triplet

Self- and oxygen-quenching processes for the triplet state of PG were investigated. The change in lifetime was traced at a wavelength of 335 nm for both types of quenching.

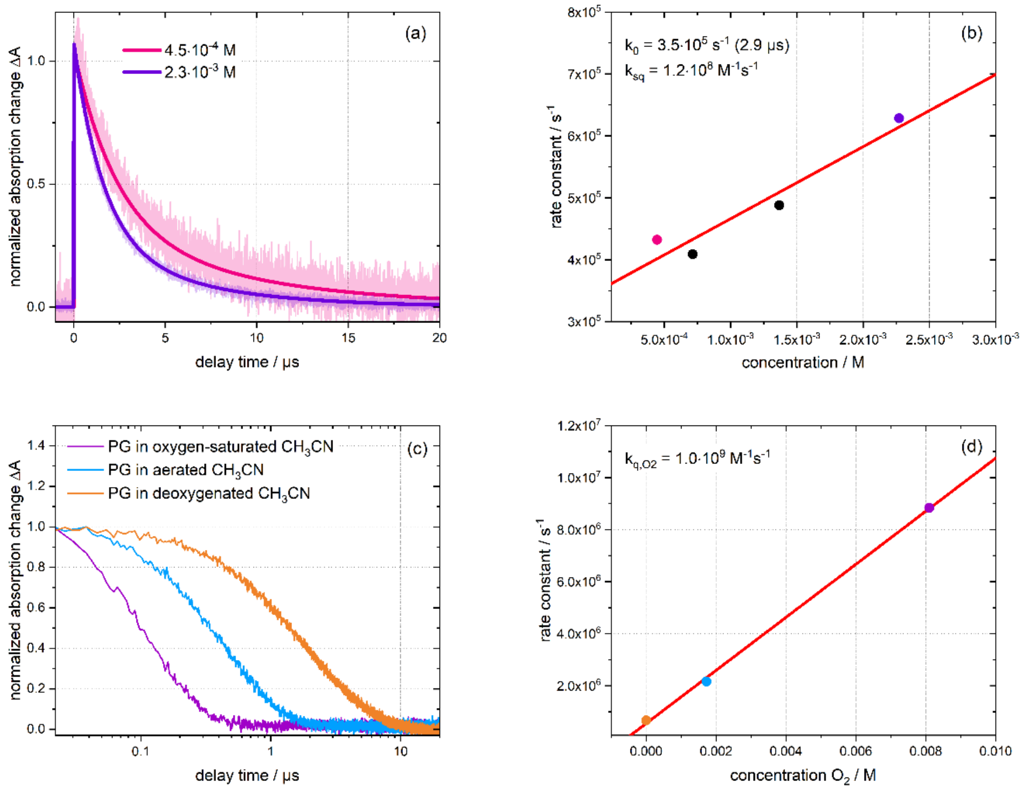

Figure S7. Bimolecular quenching experiments: (a) Triplet absorption change as a function of the delay time for a low ($7.1{\cdot}10^{-4}$ M) and high ($2.3{\cdot}10^{-3}$ M) PG concentration in deoxygenated CH_3CN. (b) Rate constants of the PG triplet decay as a function of the PG concentration. The slope of this plot yields a self-quenching constant k_{sq} of $1.2{\cdot}10^{8}$ $M^{-1}s^{-1}$. Its intercept equals the rate constant of the intrinsic decay k_0 of $3.5{\cdot}10^{5}$ s^{-1} (c) Triplet decay traces for different oxygen concentrations (PG concentration of $9.6{\cdot}10^{-4}$ M). (d) Rate constants of the PG triplet decay as a function of the O_2 concentration. These concentrations correspond to partial oxygen pressures of 0 bar, 0.213 bar and 1.013 bar.[1] The slope of this plot yield rate constant for oxygen-quenching $k_{q,O2}$ of $1.0{\cdot}10^{9}$ $M^{-1}s^{-1}$.

4. Time-Resolved IR Spectroscopy

Femtosecond and nanosecond experiments on phosphate buffered *m*-nitrophenylacetic acid were performed. The CO_2 signal at 2339 cm^{-1} increases with a time constant of ~ 170 ps, which is close to the published value.[2] In the nanosecond experiment, the CO_2 signal rises within the instrumental response time. It should be mentioned that the sample precipitated during the nanosecond IR measurement. The signal-to-noise ratio is therefore lower than with the PG measurement.

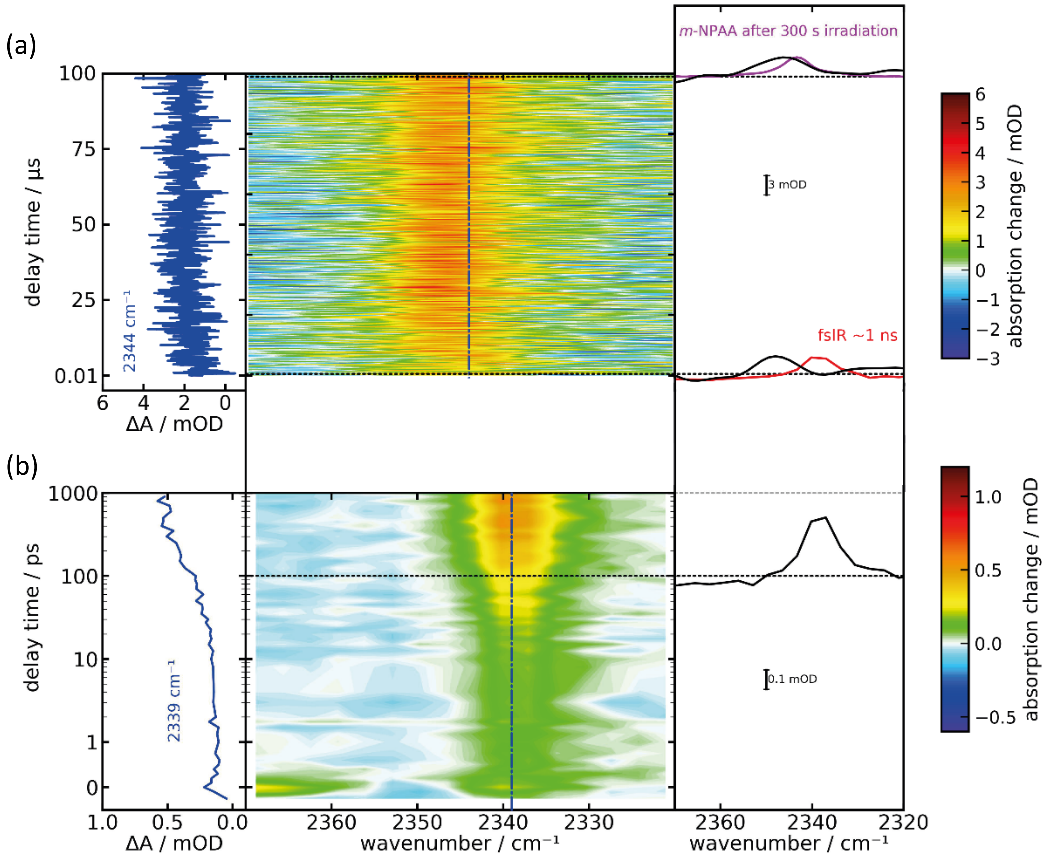

Figure S8. Femtosecond (a) and nanosecond (b) IR spectroscopy on *m*-nitrophenylacetic acid dissolved in sodium phosphate buffer (100 mM, pH 7.4). In the central contour representation, the difference absorption as a function of detection wavelength and delay time is color-coded. Vertical lines mark spectral positions for the time traces plotted on the left. Horizontal lines mark delay times for the difference spectra plotted on the right. In the femtosecond experiment, the excitation was tuned to 296 nm and the concentration amounted to 10 mM measured in a 100 µm flow cell. In the nanosecond experiment, the excitation wavelength was 266 nm and the concentration amounted to 19 mM measured in a 47 µm flow cell. For the nanosecond experiment, the phosphate buffered sample was deoxygenated.

5. Quantum Chemical Calculations

The ground state molecular geometry of the conformers (open and closed) of PG were geometry-optimized with acetonitrile as solvent using the polarizable continuum model (PCM) (SCRF method). The gradient-corrected exchange-correlation B3LYP density functional and Gaussian type Def2-TZVP basis functions were used.[3] The computations were performed with Gaussian16.[4]

Table S1. Atomic coordinates for the open as well as the closed conformer of PG. Both conformers are optimized in the electronic ground state in acetonitrile at the B3LYP/Def2-TZVP level of theory.

	**open conformer**				**closed conformer**		
	x	y	z		x	y	z
C	-3.71199	0.69676	0.38665	C	-3.65050	-0.95307	-0.26921
C	-2.54267	1.41850	0.13648	C	-2.40180	-1.52437	-0.01282
C	-1.39149	0.69633	-0.10953	C	-1.32912	-0.66493	0.11971
C	-3.71158	-0.69834	0.38624	C	-3.79988	0.42833	-0.39066
C	-2.54185	-1.41926	0.13564	C	-2.70654	1.28846	-0.25878
C	-1.39109	-0.69628	-0.10995	C	-1.47784	0.71494	-0.00083
H	-4.63394	1.22708	0.58510	H	-4.51682	-1.59196	-0.37673
H	-4.63323	-1.22931	0.58438	H	-4.77994	0.84007	-0.59140
H	-2.53731	2.50001	0.13619	H	-2.27922	-2.59478	0.07934
H	-2.53587	-2.50076	0.13471	H	-2.81715	2.35987	-0.35477
C	-0.01126	1.16199	-0.40030	C	0.09974	-0.94885	0.37228
C	-0.01060	-1.16096	-0.40102	C	-0.14760	1.34556	0.19152
N	0.74779	0.00079	-0.58499	N	0.74927	0.27600	0.40686
O	0.42472	2.28764	-0.47773	O	0.66304	-2.02423	0.50369
O	0.42605	-2.28631	-0.47919	O	0.16369	2.51046	0.18788
C	2.16388	0.00120	-0.81403	C	2.14450	0.46164	0.76741
C	2.95766	-0.00037	0.49100	C	3.14401	-0.03716	-0.28026
H	2.43088	0.88705	-1.39205	H	2.34568	-0.06177	1.70546
H	2.43113	-0.88302	-1.39441	H	2.30735	1.52490	0.91246
O	4.29289	-0.00047	0.35017	O	3.02052	-1.31137	-0.65817
O	2.45927	-0.00144	1.58338	O	4.02470	0.66015	-0.71568
H	4.56631	0.00050	-0.57913	H	2.26965	-1.75164	-0.19425

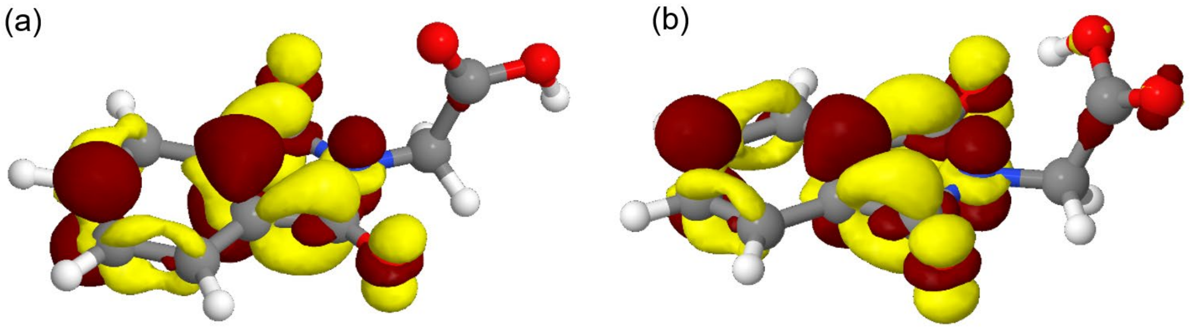

Figure S9. Electron density difference isosurface map with the isovalue 0.002 atomic units between the T_1 and ground state for the T_1 geometry; a) open conformer and b) closed conformer (yellow, electron density increases upon transition; dark red electron density decreases upon transition). The maps were constructed with Gaussian16 cubegen.

In the time-resolved nsIR measurement of PG (73 mM) in deoxygenated acetonitrile, the signatures of the triplet (1646 cm^{-1} and 1689 cm^{-1}; ~0.3 µs) and the CO_2 (2343 cm^{-1}; ~9 µs) can be clearly assigned. Further signatures between 8 µs and 10 µs are recognizable. Due to the high concentration used, these could be signatures of the ketyl and carboxylate radical. To support this hypothesis, quantum chemical calculations were carried out using Gaussian16[4] (UB3LYP/6-311++G(d,p) level of theory). The solvent acetonitrile was considered implicitly using the polarizable continuum model (PCM, SCRF method). A fundamental scaling factor of 0.9688[5] was applied to all calculated frequencies. The computed signature is compared with the respective measurement (cf. Figure 5). For a synthetic difference spectrum of the radicals (Figures S10), the sum of the respective convoluted Gaussians (ketyl and carboxyl radical; FWHM ~17 cm^{-1}) was calculated and the calculated ground state was subtracted twice. For the ketyl radical, a weak vibration of the phenyl ring is predicted at 1567 cm^{-1} and the symmetric CO stretching vibration of the imide at 1599 cm^{-1}. At 1685 cm^{-1} the vibration of the anti-symmetric CO stretching vibration is predicted for the carboxylate radical. The similarity of the computed and experimental patterns lends credit to the postulated *inter*molecular mechanism.

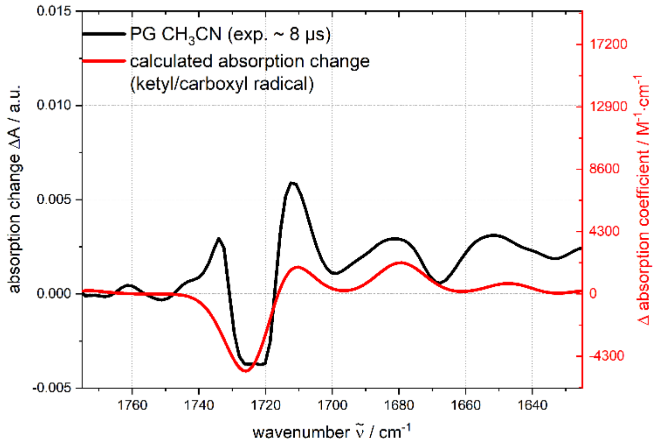

Figure S10. Synthetic difference IR spectrum based on the sum of ketyl and carboxyl radical (scaling factor 0.9688[5]) in comparison with the nsIR PG in deoxygenated acetonitrile in the average time range of 8 µs - 10 µs. The calculated IR spectra were shifted +30 cm^{-1} (+80 cm^{-1}) for the carboxyl radical (ketyl radical).

References

1. J. Achord and C. Hussey, *Anal. Chem.*, 1980, **52**, 601-602.
2. K. Neumann, M. K. Verhoefen, J. M. Mewes, A. Dreuw and J. Wachtveitl, *Phys. Chem. Chem. Phys.*, 2011, **13**, 17367-17377.
3. M. K. Kesharwani, B. Brauer and J. M. L. Martin, *J. Phys. Chem. A*, 2015, **119**, 1701-1714.
4. G. W. T. M. J. Frisch, H. B. Schlegel, G. E. Scuseria, M. A. Robb, J. R. Cheeseman, G. Scalmani, V. Barone, G. A. Petersson, H. Nakatsuji, X. Li, M. Caricato, A. V. Marenich, , B. G. J. J. Bloino, R. Gomperts, B. Mennucci, H. P. Hratchian, J. V. Ortiz, A. F. Izmaylov, J. L. Sonnenberg, D. Williams-Young, F. Ding, F. Lipparini, F. Egidi, J. Goings, B. Peng, A. Petrone, T. Henderson, D. Ranasinghe, V. G. Zakrzewski, J. Gao, N. Rega, , W. L. G. Zheng, M. Hada, M. Ehara, K. Toyota, R. Fukuda, J. Hasegawa, M. Ishida, T. Nakajima, Y. Honda, O. Kitao, H. Nakai, and K. T. T. Vreven, J. A. Montgomery, Jr., J. E. Peralta, F. Ogliaro, M. J. Bearpark, J. J. Heyd, E. N. Brothers, K. N. Kudin, V. N. Staroverov, T. A. Keith, R. Kobayashi, J. Normand, K. Raghavachari, A. P. Rendell, J. C. Burant, S. S. Iyengar, J. Tomasi, M. Cossi, J. M. Millam, M. Klene, C. Adamo, R. Cammi, J. W. Ochterski, R. L. Martin, K. Morokuma, O. Farkas, J. B. Foresman, and D. J. Fox, *Gaussian 16, Revision A.03*, Gaussian, Inc., Wallingford CT, 2016.
5. J. P. Merrick, D. Moran and L. Radom, *J. Phys. Chem. A*, 2007, **111**, 11683-11700.
